# A subcortical switchboard for perseverative, exploratory and disengaged states

**DOI:** 10.1038/s41586-025-08672-1

**Published:** 2025-03-05

**Authors:** Mehran Ahmadlou, Maryam Yasamin Shirazi, Pan Zhang, Isaac L. M. Rogers, Julia Dziubek, Margaret Young, Sonja B. Hofer

**Affiliations:** Sainsbury Wellcome Centre, University College London, London, UK

**Keywords:** Neural circuits, Neuroscience

## Abstract

To survive in dynamic environments with uncertain resources, animals must adapt their behaviour flexibly, choosing strategies such as persevering with a current choice, exploring alternatives or disengaging altogether. Previous studies have mainly investigated how forebrain regions represent choice costs and values as well as optimal strategies during such decisions^1–5^. However, the neural mechanisms by which the brain implements alternative behavioural strategies such as persevering, exploring or disengaging remain poorly understood. Here we identify a neural hub that is critical for flexible switching between behavioural strategies, the median raphe nucleus (MRN). Using cell-type-specific optogenetic manipulations, fibre photometry and circuit tracing in mice performing diverse instinctive and learnt behaviours, we found that the main cell types of the MRN—GABAergic (γ-aminobutyric acid-expressing), glutamatergic (VGluT2^+^) and serotonergic neurons—have complementary functions and regulate perseverance, exploration and disengagement, respectively. Suppression of MRN GABAergic neurons—for instance, through inhibitory input from lateral hypothalamus, which conveys strong positive valence to the MRN—leads to perseverative behaviour. By contrast, activation of MRN VGluT2^+^ neurons drives exploration. Activity of serotonergic MRN neurons is necessary for general task engagement. Input from the lateral habenula that conveys negative valence suppresses serotonergic MRN neurons, leading to disengagement. These findings establish the MRN as a central behavioural switchboard that is uniquely positioned to flexibly control behavioural strategies. These circuits thus may also have an important role in the aetiology of major mental pathologies such as depressive or obsessive-compulsive disorders.

## Abstract (web summary)

Behavioural experiments in mice demonstrate that GABAergic (γ-aminobutyric acid-expressing), glutamatergic and serotonergic neurons in the median raphe nucleus have distinct and complementary functions in regulating decision-making resulting in flexible behavioural strategies.

## Main

**Extended Data Fig. 1:**
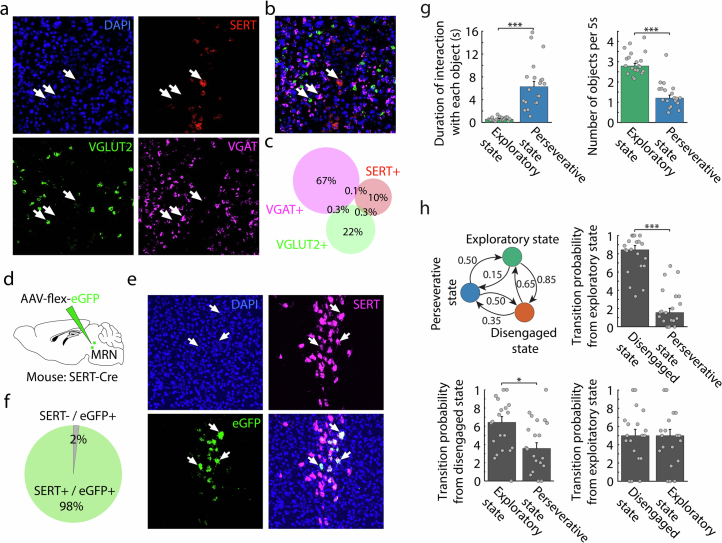
Expression and overlap of VGAT, VGluT2 and SERT in MRN, and interaction states extracted by hidden Markov model. **a**, Example image of multi-colour single molecule in-situ mRNA hybridization in MRN. DAPI is shown in blue, and SERT+, VGAT+, and VGluT2+ cells are in red, magenta, and green, respectively. White arrows indicate the location of example cells. **b**, Overlap between the four colours of the example slice shown in **a**. **c**, Venn diagram of MRN cells expressing SERT, VGAT and VGluT2, shown in percentages of all counted MRN neurons (*N* = 620 neurons from 5 slices, 3 mice). **d**, Schematic of experimental design to express eGFP in Cre-expressing cells in the MRN of SERT-Cre mice. **e**, Example of multi-colour single molecule in-situ mRNA hybridization in MRN. DAPI is shown in blue, and SERT+ and eGFP+ cells in magenta and green, respectively. White arrows indicate the location of example cells. The bottom right panel shows the overlap between the three colours. **f**, Venn diagram of SERT+ and SERT- MRN cells expressing eGFP, shown in percentage of counted neurons (*N* = 51 neurons from 4 slices, 3 mice). **g**, Average duration of object interactions (left, P = 2.3 × 10^−6^, two-sided paired t-test; N = 20 experiments from 10 mice) and frequency of object interaction (right, P = 0.0001, two-sided Wilcoxon signed rank test) in exploratory (green) and perseverative (blue) states in control mice during the MNOI test. **h**, Median transition probabilities at the time of switching between the three interaction states in control mice. Bar graphs show the transition probability from each state to the other two states (median values and individual experiments). From exploratory state to disengaged vs. perseverative state: P = 0.0003, two-sided Wilcoxon signed rank test; from disengaged state to exploratory vs. perseverative state: P = 0.0302, two-sided paired t-test; from perseverative state to disengaged state vs. exploratory state: P = 0.8047, two-sided paired t-test, N = 20 experiments from 10 mice). *: p-value < 0.05, ***: p-value < 0.001. Bars depict median, error bars are bootstrapped standard error and circles indicate individual experiments.

Animals are adept at switching their modus operandi to adjust to changes in environmental conditions, the availability of resources and internal needs. At any moment in time, they have to decide between competing strategies that govern their interactions with environmental resources, such as whether to explore, to exploit or to disengage from the environment. Exploitation—the efficient utilization of known resources, for instance, through perseverance in familiar choices—ensures immediate gains and minimizes risk. By contrast, exploration entails the more labour-intensive endeavour of seeking out novel opportunities and gathering knowledge to increase the chance of future gains^6,7^. Alternatively, animals can disengage from active pursuit of goals with the benefit of conserving energy and minimizing exposure to predator risk. Previous studies on the neural basis of explore–exploit decisions have focused primarily on the role of prefrontal cortical areas (PFC) in evaluating the costs and computing the anticipated value of different choices^1–5^, and on the role of dopaminergic signalling in striatum and PFC for updating expected choice value^3,8,9^. However, maintaining the correct balance between exploratory, perseverative and disengaged states is crucial for survival across the animal kingdom, indicating that the neural circuits that underpin these behavioural strategies are evolutionarily conserved^10–13^. Although the identity of these circuits has remained unknown, we speculated that they must encompass subcortical neural pathways that enable animals to maintain or switch between behavioural strategies independently of higher-order cognitive functions and telencephalic circuits. In this study, we identify the MRN in the brainstem as a key switchboard for controlling perseverative behaviour, exploration and disengagement across instinctive and acquired behaviours. The MRN provides a neural nexus at the interface of internal state, affective and cognitive information from brain regions such as the hypothalamus, habenula and PFC^14–17^. Alongside the dorsal raphe nucleus (DRN), it is a main source of the neuromodulator serotonin, which has been broadly implicated in behavioural flexibility, avoidance, perseverative behaviour and obsessive disorders^13,18–22^. Although most previous studies have focused on dorsal raphe serotonergic signalling, serotonergic neurons specifically in the MRN have also been suggested to have a role in sustainment of goal-directed behaviour and avoidance^22–24^. However, the MRN does not only contain serotonergic neurons—only 5–10% of MRN neurons are serotonergic^25,26^, whereas the majority are GABAergic^26^ (VGAT^+^, around 60%) or glutamatergic (VGluT2^+^, around 25% (ref. ^26^) and VGluT3^+^, mainly overlapping with SERT^+^ (ref. ^27^)). Consistent with previous studies, we found that GABAergic, serotonergic and VGLuT2-expressing neurons in the MRN constitute separate cell classes with minimal overlap^26^ (Extended Data Fig. 1a–c).

## VGAT^+^ MRN neurons regulate perseverance

**Fig. 1:**
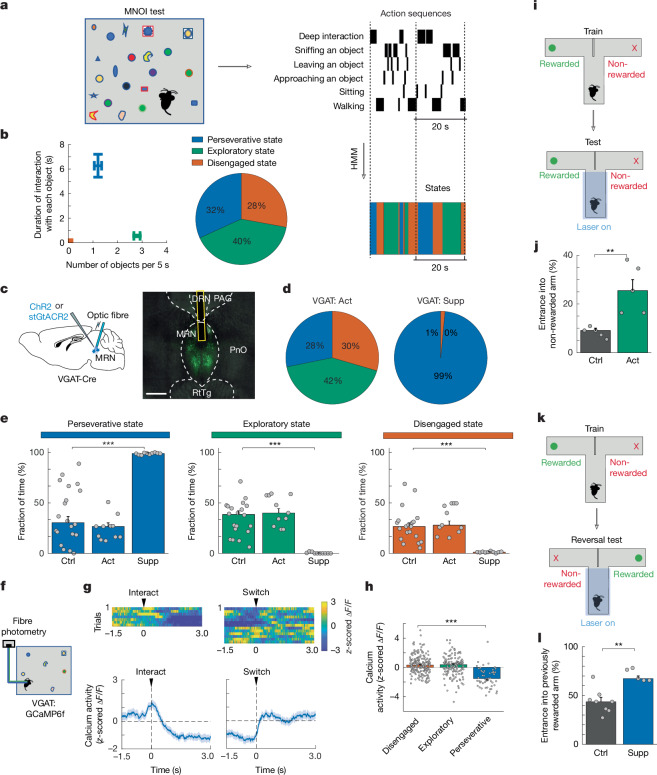
VGAT-expressing MRN neurons control perseverative state. **a**, Schematic of the MNOI test (left) and an example sequence of annotated actions (top right), with three interaction states extracted by a HMM (bottom right). **b**, Left, average duration of interaction with each object plotted against the frequency of interactions with different objects in each HMM state in control mice (median ± bootstrap standard error, *n* = 20 experiments from 10 mice). Right, median fraction of time spent in each state. **c**, Left, schematic of optogenetically activating or suppressing VGAT^+^ MRN neurons. Right, example image of virus expression in VGAT^+^ MRN neurons with optic fibre position. PAG, periaqueductal grey; PnO, pontine reticular formation; RtTg, reticulotegmental nucleus. Scale bar, 0.5 mm. **d**, Median fraction of time spent in the perseverative (blue), exploratory (green) and disengaged state (orange) during the MNOI test in mice with activation (Act; left) or suppression (Supp; right) of VGAT^+^ MRN neurons. **e**, Fraction of time spent in each interaction state in tdTomato control mice (ctrl) and mice shown in **d** with activation or suppression of VGAT^+^ MRN neurons. Bars indicate median values, error bars are bootstrapped standard error and circles represent individual experimental sessions. *n* = 20, 11 and 10 experiments from 10, 6 and 5 mice in tdTomato, VGAT^+^ neuron activation and VGAT^+^ suppression groups, respectively. **f**, Schematic of calcium fibre photometry recording from VGAT^+^ MRN neurons in mice exposed to multiple novel objects. **g**, Heat map of *z*-scored calcium activity traces of an example experiment (top) and average *z*-scored calcium activity trace (bottom; mean ± s.e.m., averaged over all events from 5 mice (*n* = 66 and 193 deep interaction and switch events)) of VGAT^+^ MRN neurons during object interactions aligned either to the onset of deep interactions (left) or the time of switching between objects (right; early example traces are from switching objects following a deep interaction, later example are traces during switching between objects without deep interactions). **h**, Median *z*-scored calcium activity (median ± bootstrap standard error) of VGAT^+^ MRN neurons during disengaged states (*n* = 190 events from 5 mice), exploratory states (*n* = 139 events from 5 mice) and perseverative states (*n* = 51 events from 5 mice). **i**, Schematic of experimental design to quantify levels of exploration during a T-maze test. During the test, in each trial photo-stimulation is applied throughout the central arm until mice turn into the left or right arm. **j**, Percentage of trials with entrance into the non-rewarded arm (median ± bootstrap standard error), indicating exploratory behaviour in tdTomato control mice (*n* = 5 mice) and mice with optogenetic activation of VGAT^+^ MRN neurons (green, 5 mice) trained in the same task. **k**, Schematic of experimental design to quantify levels of perseverative behaviour during a reversal test in the T-maze. **l**, Percentage of trials with entrance into the previously rewarded (but now non-rewarded) arm during the reversal T-maze test (median ± bootstrap standard error) in tdTomato control mice (*n* = 8 mice) compared with mice with optogenetic suppression of VGAT MRN neurons (blue, 5 mice). *P < 0.05; **P < 0.01; ***P < 0.001. See Extended Data Table 1 for statistics. Source data

To test whether any of these three main MRN cell types have a role in regulating animals’ natural behavioural strategies for interacting with the environment, we aimed to establish a paradigm in which mice display perseverative, exploratory and disengaged behavioural interaction states during instinctive, naturalistic behaviour without the need for prior knowledge or training. Freely moving mice were exposed to 20 small, novel objects, and their behaviour was captured on video and scored (Fig. 1a and Supplementary Video 1). Labelled actions during this multi-novel object interaction (MNOI) test included actions attributed to a specific object, such as approaching and leaving an object, deep interaction (defined as the mouse grabbing, carrying or biting the object) and shallow interaction (defined as the mouse sniffing or in close contact with an object and then leaving without deep interaction), as well as sitting and walking without object interaction. We trained an unsupervised hidden Markov model (HMM) on control mice to categorize the sequences of labelled actions into three interaction states (Methods). In periods assigned to state 1 by the HMM, mice were mainly engaged in deep, long-duration interactions with one or few objects. We named this state in which mice showed such sustained actions the perseverative state. In state 2, mice exhibited rapid switching between several objects without deep interactions—this state was therefore named the exploratory state. In state 3, the disengaged state, mice were passive or walked around without object interaction (Fig. 1b, Extended Data Fig. 1g,h and Supplementary Video 1). Control mice spent roughly equal amounts of time in each of these three states (Fig. 1b).

**Extended Data Fig. 2:**
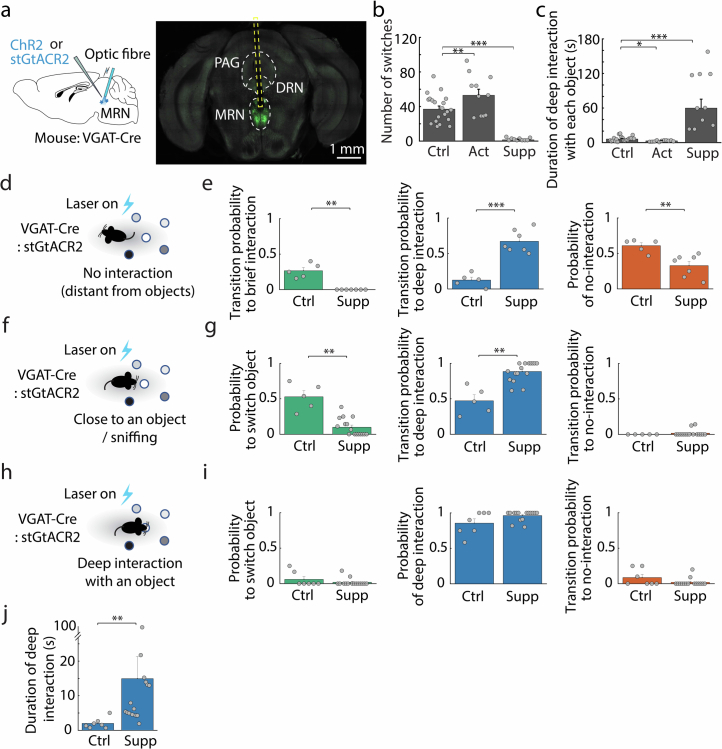
Impact of tonic and phasic optogenetic stimulation of VGAT+ MRN neurons on object interaction. **a**, Schematic of experimental design: optogenetic activation or suppression of VGAT+ MRN neurons, using ChR2 or stGtACR2 (left) and example image of virus expression in VGAT+ MRN neurons with optic fibre position (right). DRN: dorsal raphe nucleus, MRN: median raphe nucleus, PAG: periaqueductal gray. **b**, Number of switches between objects during the MNOI test in control (ctrl) mice and mice with activation (act) and suppression (supp) of VGAT + MRN neurons (ctrl vs. act: P = 0.00591, ctrl vs. supp: P = 2.3 × 10^−7^, two-sided t-test with Bonferroni multi-comparison correction). N = 20, 11 and 10 experiments from 10, 6 and 5 mice in control, VGAT+ activation and VGAT+ suppression groups. **c**, Duration of deep interactions (when mice grab, bite or carry the object) with each object during the MNOI test of mice in **b** (ctrl vs. act: P = 0.0102, ctrl vs. supp: P = 1.6 × 10^−6^, two-sided t-test with Bonferroni multi-comparison correction). **d**, Schematic of the experimental design to examine the effect of phasic 2-second suppression of VGAT+ MRN neurons in the MNOI test (with 5 objects) when the animal was not interacting with an object. **e**, Transition probability from no object interaction within the 2-second stimulation window (in experiment in **d**) to brief interaction (left, tdTomato ctrl (N = 5 experiments from 5 mice) vs VGAT+ supp. (N = 7 experiments from 7 mice), P = 0.0025; two-sided Mann-Whitney U test), to deep interaction (middle, ctrl vs VGAT+ supp.: P = 4.2 × 10^−5^; two-sided t-test), and probability to remain not interacting with the objects (right, ctrl vs VGAT+ supp.: P = 0.0054; two-sided t-test). **f**, Phasic 2-second suppression of VGAT+ MRN neurons in the MNOI test when the animal’s snout is close to an object. **g**, Probability within the 2-second stimulation window (in experiment in **f**) to switch to a different object (left, ctrl (N = 5 experiments from 5 mice) vs VGAT+ supp. (N = 16 experiments from 7 mice), P = 0.0012; two-sided Mann-Whitney U test), to transition to a deep interaction (middle, ctrl vs VGAT+ supp.: P = 0.0016; two-sided Mann-Whitney U test), and to transition to no-interaction (right, ctrl vs VGAT+ supp.: P = 0.4660; two-sided Mann-Whitney U test). **h**, Phasic 2-second suppression of VGAT+ MRN neurons in the MNOI test during deep interaction with an object. **i**, Probability (in experiment in **h**) to switch object (left, ctrl (N = 7 experiments from 5 mice) vs VGAT+ supp. (N = 15 experiments from 7 mice), P = 0.3733; two-sided Mann-Whitney U test), to persist in deep interaction (middle, ctrl vs VGAT+ supp.: P = 0.0857; two-sided Mann-Whitney U test), and to transition to no-interaction (right, ctrl vs VGAT+ supp.: P = 0.1025; two-sided Mann-Whitney U test). **j**, Median duration of deep interactions after 2-second laser stimulation during deep object interactions (in experiments in **h**, in ctrl vs VGAT+ supp.: P = 0.0015; two-sided Mann-Whitney U test). *: p-value < 0.05, **: p-value < 0.01, ***: p-value < 0.001. In **b, c, e, g, i** and **j** bars depict mean, error bars are standard error and circles indicate individual experiments.

**Extended Data Fig. 3:**
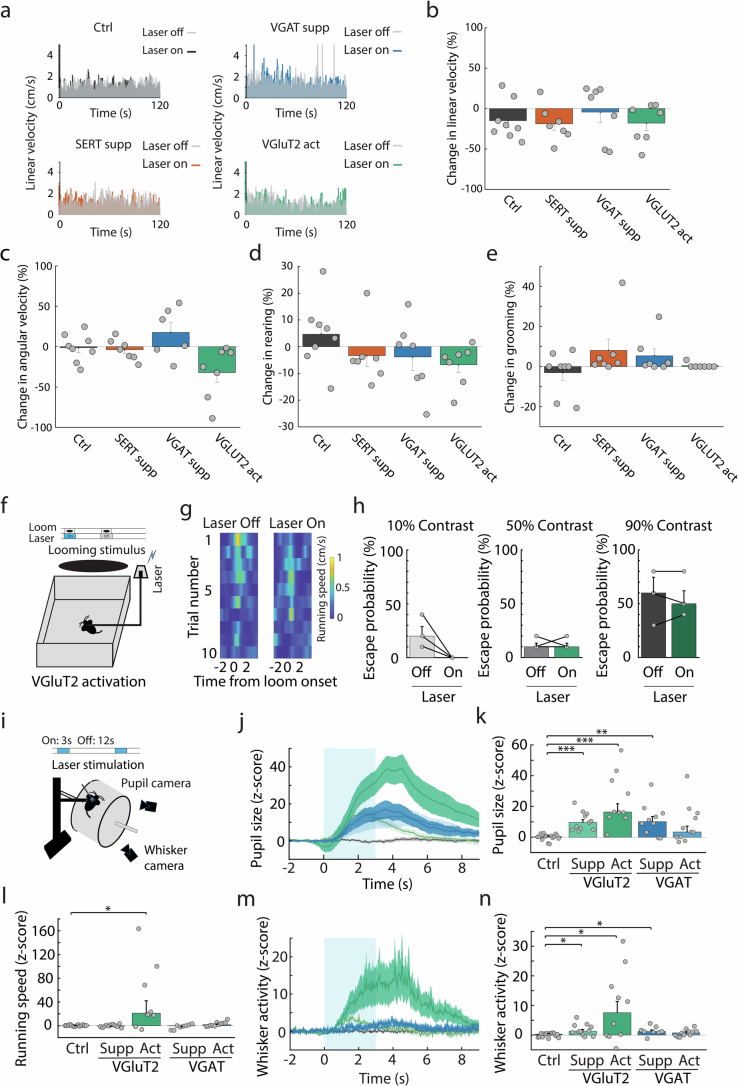
Effect of MRN cell type manipulation on motor behaviour in an open field, and on arousal and valence. **a**, Linear velocity over the course of a 2-minute open field test in laser-on and laser-off conditions, averaged over mice in a tdTomato control group (ctrl; N = 8 mice), and in groups of mice with suppression of SERT+ (SERT+ supp; N = 7 mice), suppression of VGAT+ (VGAT+ supp; N = 7 mice), and activation of VGluT2+ (VGluT2+ act; N = 7 mice) MRN neurons. **b**, Optogenetic stimulation effect on average linear velocity over time (100 × (linear velocity during laser-on period - linear velocity during laser-off period)/linear velocity during laser-off period) in mice in **a**. Control mice (N = 8) are compared to SERT+ supp (N = 7 mice, P > 0.9999), VGAT+ supp (N = 7 mice, P > 0.9999) and VGluT2+ act (N = 7 mice, P > 0.9999); two-sided t-test with Bonferroni multi-comparison correction. **c**, Optogenetic stimulation effect on averaged angular velocity over time (100 × (angular velocity during laser-on period - angular velocity during laser-off period)/angular velocity during laser-off period) in mice in **a**. Control mice are compared to SERT+ supp (P > 0.9999), VGAT+ supp (P = 0.5144) and VGluT2+ act (P = 0.1110); two-sided t-test with Bonferroni multi-comparison correction. **d**, Optogenetic stimulation effect on time spent rearing (100 × (time spent rearing in laser-on period – time spent rearing in laser-off period)/total test duration) in mice in **a**. Control mice are compared to mice with SERT+ supp (P = 0.6436), VGAT+ supp (P = 0.6800) and VGluT2+ act (P = 0.1739); two-sided t-test with Bonferroni multi-comparison correction. **e**, Optogenetic stimulation effect on time spent grooming (100 × (time spent grooming in laser-on period – time spent grooming in laser-off period)/total test duration) in mice in **a**. Control mice are compared to mice with SERT+ supp (P = 0.3647), VGAT+ supp (P = 0.3885) and VGluT2+ act (P > 0.9999); two-sided t-test with Bonferroni multi-comparison correction. In **b-e**, bars depict mean, error bars are standard error and circles indicate individual experiments. **f**, Schematic of the experimental design for quantifying fear responses to an innately aversive dark looming stimulus, with three different stimulus contrast levels (10%, 50%, and 90%), with and without optogenetic activation of VgluT2+ MRN neurons. **g**, Heatmaps show running speed (cm/s) of an example mouse in response to 90% contrast looming stimuli in laser off and laser on trials. **h**, Escape probability of mice in response to looming stimuli with different contrasts, during activation of VgluT2+ MRN neurons (laser on) and control trials (laser off). N = 3 mice, 10% contrast: P = 0.1181; 50% contrast: P > 0.9999; 90% contrast: P > 0.9999, two-sided paired t-test. **i**, Schematic of the experimental design to examine the effects of optogenetic manipulation of VGluT2+ and VGAT+ MRN neurons on running speed and arousal level, measured using pupil size and whisker activity. **j**, Z-scored running speed evoked by optogenetic stimulation in control mice and mice with activation or suppression of VGluT2+ or VGAT+ MRN neurons. N = 15, 11, 9, 8 and 11 mice, respectively; P > 0.9999, P = 0.0229, P = 0.3880 and P = 0.1210 for comparing control mice to activation or suppression of VGluT2+ neurons, and activation or suppression of VGAT+ neurons, respectively; two-sided t-test with Bonferroni multi-comparison correction. **k**, Z-scored pupil size over time (mean ± s.e.m.) in control mice (grey) and mice with activation or suppression of VGluT2+ and VGAT+ MRN neurons, averaged over trials and aligned to onset of optogenetic manipulation. Light blue box indicates the laser stimulation period. **l**, Median z-score of pupil size during laser stimulation from traces in **k**. N = 15, 11, 10, 10 and 11 mice; P = 8.0 × 10^−6^, P = 6.7 × 10^−5^, P = 0.0015 and P = 0.0660 for comparing control mice to activation or suppression of VGluT2+ neurons, and activation or suppression of VGAT+ neurons, respectively; two-sided t-test with Bonferroni multi-comparison correction. **m,n**, same as **k,l** but for z-scored whisker activity (summation of absolute frame-by-frame differences in pixel luminance). N = 15, 11, 10, 8 and 11 mice, respectively; P = 0.0165, P = 0.0195, P = 0.0153 and P = 0.2356 for comparing control mice to activation or suppression of VGluT2+ neurons, and activation or suppression of VGAT+ neurons, respectively; two-sided t-test with Bonferroni multi-comparison correction. *: p-value < 0.05, **: p-value < 0.01, ***: p-value < 0.001. Bars in **h, k, l** and **n** depict median, error bars are bootstrapped standard error and circles indicate individual experiments.

Because they comprise the largest fraction of neurons in the MRN^26^, we first investigated the potential role of VGAT-positive MRN (MRN^VGAT^) neurons in regulating interaction states of mice. To test whether manipulating the activity of MRN^VGAT^ neurons changes the time mice spend in a perseverative, exploratory or disengaged state, we expressed either a Cre-dependent soma-targeted inhibitory opsin, stGtACR2, or an excitatory opsin, ChR2, in MRN^VGAT^ neurons of VGAT-Cre mice via carefully targeted viral injections (Fig. 1c and Extended Data Fig. 2a), to optogenetically suppress or activate these neurons, respectively, during the 2-min duration of the MNOI test. Suppression of MRN^VGAT^ neurons induced a marked increase in sustained interactions with individual objects, with far fewer switches between objects as compared with control mice expressing tdTomato in MRN neurons (Fig. 1d,e, Extended Data Fig. 2b,c and Supplementary Video 2). Mice thus spent most time in what we defined as the perseverative state when MRN^VGAT^ neurons were suppressed, and, consequently, the duration of both exploratory and disengaged states decreased to near zero (Fig. 1d,e). Basic motor behaviour without the presence of objects was not affected by this manipulation (Extended Data Fig. 3a–e). Activation of MRN^VGAT^ neurons in the MNOI test did not result in a significant change in the duration of the three interaction states (Fig. 1d,e). It did, however, significantly decrease how long mice deeply interacted with individual objects and increase how often they switched between objects, suggesting that activation of MRN^VGAT^ neurons may suppress sustained interactions (Extended Data Fig. 2b,c). Together, these results show that continual manipulation of MRN^VGAT^ neurons can strongly bias mouse behaviour. To test whether a short change in activity can also cause a real-time behavioural shift, we suppressed MRN^VGAT^ neurons for only 2 s during specific behavioural epochs in the MNOI test—when mice were not engaged with an object, had just started interacting, or were deeply interacting with an object. In all cases, brief MRN^VGAT^ neuron suppression induced persistent object interaction (Extended Data Fig. 2d–j).

To determine whether MRN^VGAT^ neurons are naturally suppressed during sustained object interactions and perseverative behaviour, we recorded population calcium signals of MRN^VGAT^ neurons (after Cre-dependent GCaMP6f expression in the MRN of VGAT-Cre mice) using fibre photometry during the MNOI test (Fig. 1f). In line with the effects of optogenetic manipulation, the activity of MRN^VGAT^ neurons was significantly suppressed throughout sustained object interactions and returned to baseline levels when mice switched to a different object (Fig. 1g), showing that these neurons are indeed engaged during this behavioural paradigm. MRN^VGAT^ neuron activity was also decreased in general during the perseverative state extracted from the HMM model, compared with exploratory and disengaged states (Fig. 1h).

**Extended Data Fig. 4:**
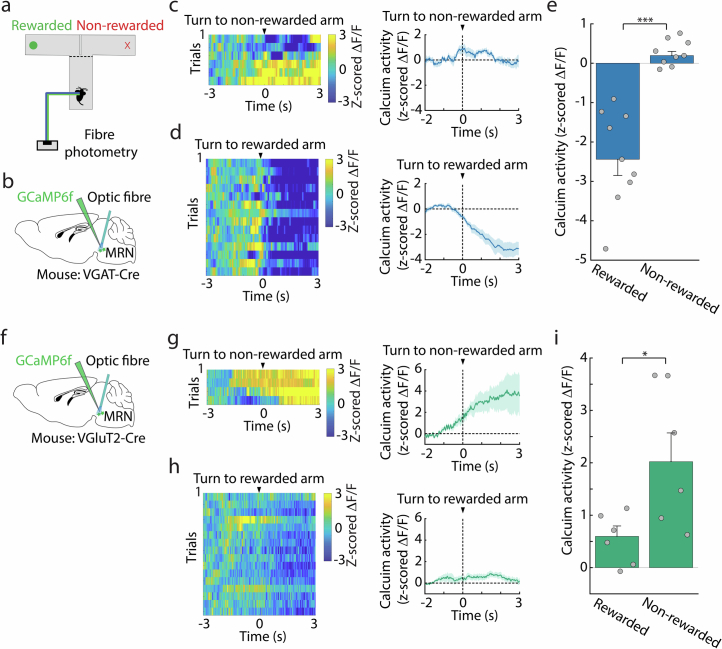
Choosing the rewarded arm suppresses VGAT+ neurons and choosing the non-rewarded arm activates VGluT2+ neurons in MRN. **a**, Schematic of calcium fibre photometry recordings from VGAT+ and VGluT2+ MRN neurons in mice during a T-maze test, after training. **b**, Schematic of the experimental design to express GCaMP6f in VGAT+ MRN neurons and implant a fibre for photometry. **c,d**, Left, Heatmap shows z-scored calcium activity traces of VGAT+ MRN neurons of an example mouse (left) and average z-scored calcium activity trace across mice (right, mean ± s.e.m., N = 9 mice) aligned to turns towards the non-rewarded arm (**c**), and aligned to turns towards the rewarded arm (**d**). **e**, Median z-scored calcium activity of VGAT+ MRN neurons during 3-second trials when the rewarded or the non-rewarded arm was chosen (N = 9 mice, P = 5.7 × 10^−5^, two-sided paired t-test). Error bars indicate bootstrapped standard error and individual data points are averaged z-scored activity over trials in individual mice. **f**, Schematic of the experimental design to express GCaMP6f in VGluT2+ MRN neurons and implant a fibre for photometry. **g**,**h**, same as **c**,**d**, but for calcium activity of VGluT2+ MRN neurons. **i**, Median z-scored calcium activity of VGluT2+ MRN neurons during trials in which the rewarded or the non-rewarded arm was chosen (N = 6 mice, P = 0.0266, two-sided paired t-test). Data point and error bars indicate median ± bootstrapped standard error. *: p-value < 0.05, ***: p-value < 0.001.

The above results show that activity of MRN^VGAT^ neurons can strongly influence how much mice persevere in an interaction during naturalistic behaviour without prior knowledge. To test whether MRN neurons can also regulate behavioural choices in tasks in which mice act on previously gained knowledge of how to maximize short-term benefits, we trained food-restricted mice on a T-maze task, in which a food reward was provided consistently only in one specific arm and not the other. In well-trained mice (over 90% correct trials in 2 sequential sessions), when MRN^VGAT^ neurons expressing ChR2 were optogenetically activated in the central arm of the T-maze, mice were more likely to choose the non-rewarded arm—that is, the exploratory option (Fig. 1j). In accordance with this result, fibre photometry recordings showed that when trained mice chose the rewarded arm, activity of MRN^VGAT^ neurons was instead consistently decreased (Extended Data Fig. 4a–e). To test the causality of this relationship and whether suppression of MRN^VGAT^ neurons biases mice towards perseverative behaviour, we reversed the reward location in the T-maze for well-trained mice—that is, the previously rewarded arm was no longer rewarded (Fig. 1k). In this scenario, control mice start exploring the other, previously non-rewarded arm and eventually learn to expect reward only at this location. When stGtACR2-expressing MRN^VGAT^ neurons were optogenetically suppressed in this reversal-learning paradigm in the central arm of the T-maze, mice remained more likely to choose the previously rewarded arm, showing an increased persistence in the previously optimal goal (Fig. 1l). This result is highly consistent with the increased duration of perseverative states caused by MRN^VGAT^ neuron suppression during the MNOI test (Fig. 1d,e), demonstrating that during both instinctive and learned goal-directed behaviours, suppression of MRN^VGAT^ neurons causes perseverance towards a current or familiar choice.

**Extended Data Fig. 5:**
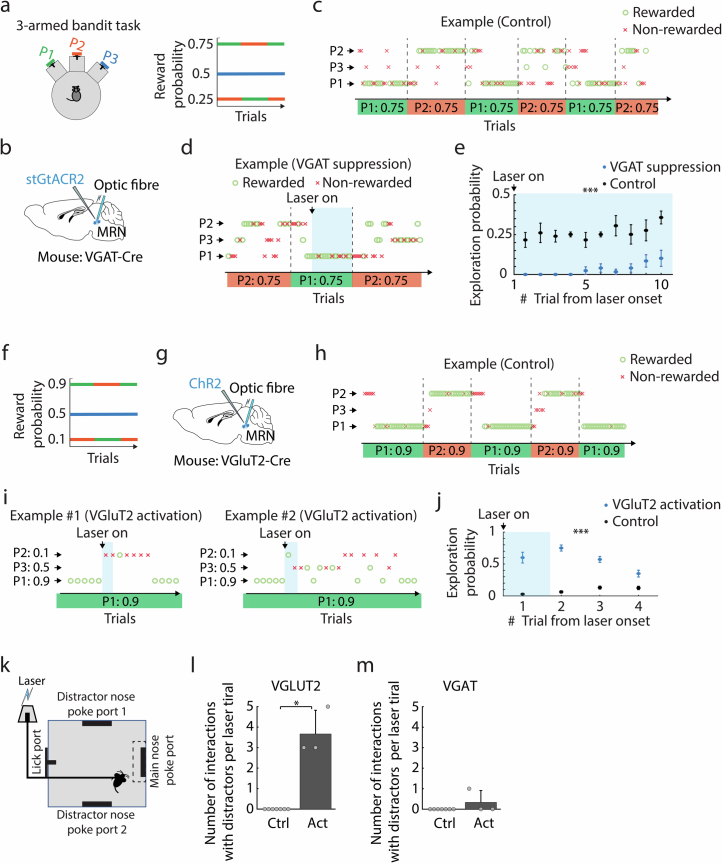
VGAT+ and VGluT2+ MRN neuron manipulation causes exploitation and exploration in multi-choice tasks. **a**, Left, Schematic of the 3-armed bandit task with lick-ports P1 to 3 to examine the effects of optogenetic suppression of VGAT + MRN neurons. Reward probability at P3 is constant at 50%, and reward probabilities at P1 and P2 fluctuate between 25% and 75% in blocks of changing lengths. **b**, Schematic of experimental design for optogenetic suppression of VGAT+ MRN neurons. **c**, Behaviour of an example VGAT-Cre mouse in a control session with no laser stimulation. X-axis shows trials and the orange and green colours indicate the blocks where P2 and P1 have the high-reward probability (0.75), respectively. Y-axis shows the chosen lick-ports and if reward was delivered (green circle) or not (red cross). **d**, Behaviour of an example mouse following suppression of VGAT+ MRN neurons during exploitation of a high-reward port (P1). Light blue bar indicates the laser stimulation period. **e**, Suppression of VGAT+ MRN neurons (blue shading indicates laser-on period) after the first occurrence of choosing the high-probability arm for 3 sequential trials in a block) decreased the animals’ exploration probability (probability of switching to one of the other two ports) during the laser-on period (blue circles), compared to control trials without laser under the same condition (black circles). N = 6 mice, P = 3.2 × 10^−4^, two-way repeated measures ANOVA (two-sided). Light blue box indicates the laser stimulation period. Data points and error bars show median ± bootstrapped standard error. **f**, Schematic of the reward probabilities in the 3-armed bandit task to examine the effects of optogenetic manipulation of VGluT2+ MRN neurons on exploration. reward probability at P3 is constant at 50%, and reward probabilities at P1 and P2 fluctuate between 10% and 90% in blocks of changing lengths. **g**, Schematic of experimental design for optogenetic activation of VGluT2+ MRN neurons. **h**, The same as **c** but for an example VGluT2-Cre mouse in a control session with reward probabilities as shown in **f** without optogenetic stimulation. **i**, Left, Examples of mouse behaviour following activation of VGluT2+ MRN neurons during exploitation of a high-reward port (P1). Light blue bar indicates the laser stimulation period. X-axis shows trials. Y-axis shows the chosen lick-ports and if reward was delivered (green circle) or not (red cross). **j**, Brief activation of VGluT2+ MRN neurons (laser on: blue circles) during a stable period of exploitation (choosing the high-probability arm for 5 sequential trials) increased the exploration probability (probability of switching to one of the other ports) over the next 4 trials, compared to control trials under the same condition (laser off: black circles). N = 11 mice, P = 6.5 × 10^−8^, two-way repeated measures ANOVA (two-sided). Light blue shading indicates the laser stimulation period. Data points and error bars show median ± bootstrapped standard error. **k**, Schematic of nose-poke reward association task with distractor nose poke ports, which have no association with reward. Mice with expression of ChR2 in VgluT2+ or VGAT+ MRN receive brief laser stimulation (1-2 s) upon entering a region of interest around the reward-associated (main) nose poke port in a subset of trials (see Methods). **l**, Median number of interactions with distractors per laser stimulation trial over all the completed laser stimulation trials in control mice and mice with optogenetic activation of VGluT2+ MRN neurons (P = 0.0027, two-sided chi-square test, N = 7 vs. 3 mice). **m**, The same as **l**, but in control mice and mice with optogenetic activation of VGAT+ MRN neurons (P > 0.9999, two-sided chi-square test, N = 7 vs. 3 mice). **: p-value < 0.01, ***: p-value < 0.001. In **l** and **m** bars depict mean, error bars are standard error and circles indicate individual experiments.

To test whether suppression of MRN^VGAT^ neurons also more generally promotes exploitatory choices, we trained mice in a three-armed bandit task with changing reward probabilities (Methods), a type of behavioural paradigm more conventionally used for studying explore–exploit trade-offs^28,29^. Indeed, when MRN^VGAT^ neurons were optogenetically suppressed, mice strongly preferred the choice with high probability of reward at the time, and ceased to explore the other options (Extended Data Fig. 5a–e). This result corroborates that this manipulation induces perseverance in exploitation of known resources.

## VGluT2^+^ MRN neurons drive exploration

**Fig. 2:**
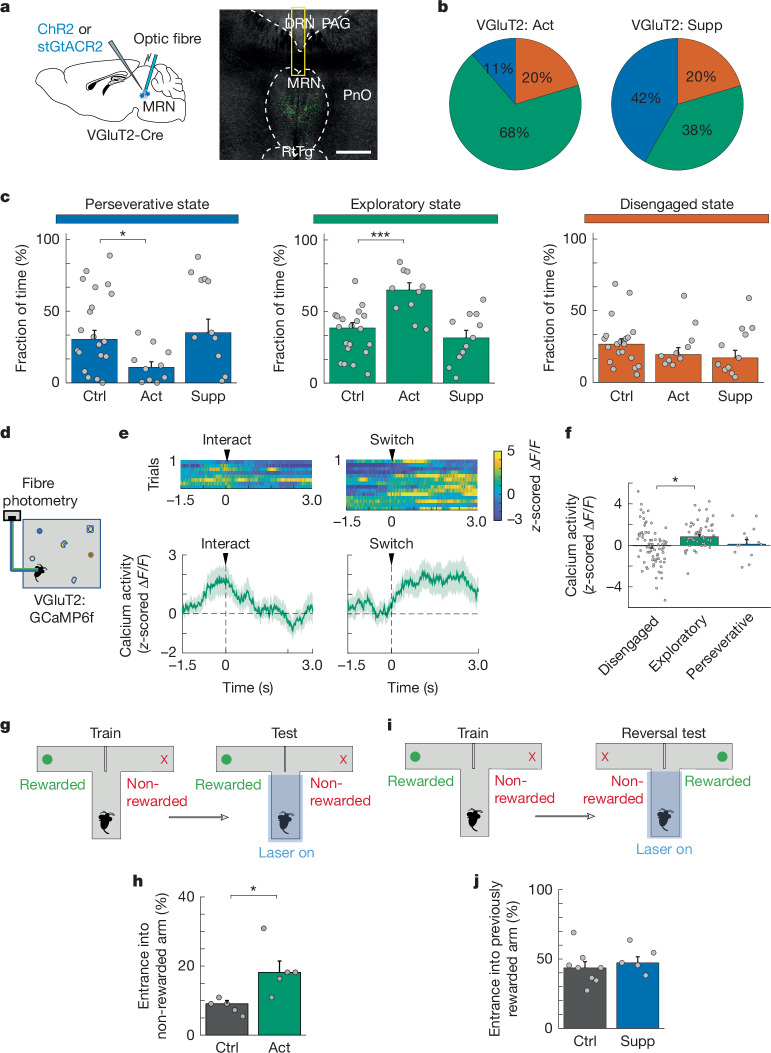
VGluT2-expressing MRN neurons drive exploratory behaviour. **a**, Left, schematic of optogenetic activation or suppression of VGluT2^+^ MRN neurons. Right, example image of virus expression in VGluT2^+^ MRN neurons with optic fibre positions. Scale bar, 0.5 mm. **b**,**c**, Median fraction of time spent in the perseverative (blue), exploratory (green) and disengaged state (orange) during the MNOI test in mice with activation (**b**, left) or suppression (**b**, right) of VGluT2^+^ MRN neurons and tdTomato control mice (**c**). *n* = 20, 10 and 11 experiments from 10, 5 and 5 mice in tdTomato, VGluT2^+^ excitation and VGluT2^+^ inhibition groups, respectively. **d**, Schematic of calcium fibre photometry recording from VGluT2^+^ MRN neurons in mice exposed to multiple novel objects. **e**, Heat map of *z*-scored calcium activity traces in an example experiment (top) and average *z*-scored calcium activity trace (bottom; mean ± s.e.m., all events from 5 mice (*n* = 17 deep interaction and 67 switch events)) of VGluT2^+^ MRN neurons aligned either to the onset of deep object interactions (left) or the time of switching between objects (right). **f**, *z*-scored calcium activity of VGluT2^+^ MRN neurons (median ± bootstrap standard error) during disengaged, exploratory and perseverative states. *n* = 72 disengaged, 53 exploratory and 13 perseverative state events from 3 mice. **g**, Experimental design to quantify levels of exploration during a T-maze test. **h**, Percentage of trials with entrance into the non-rewarded arm during the T-maze test, in tdTomato control mice (*n* = 5 mice) and mice with activation of VGluT2^+^ MRN neurons (left; 5 mice). **i**, Experimental design to quantify perseverance during a reversal test in the T-maze. **j**, Percentage of trials with entrance into the previously rewarded arm in tdTomato control mice (*n* = 8 mice) and mice with suppression of VGluT2^+^ MRN neurons (left; 5 mice). *P < 0.05; ***P < 0.001. In **c**,**h**,**j**, bars indicate median, error bars are bootstrapped standard error and circles represent individual experimental sessions. See Extended Data Table 1 for statistics. Source data

**Extended Data Fig. 6:**
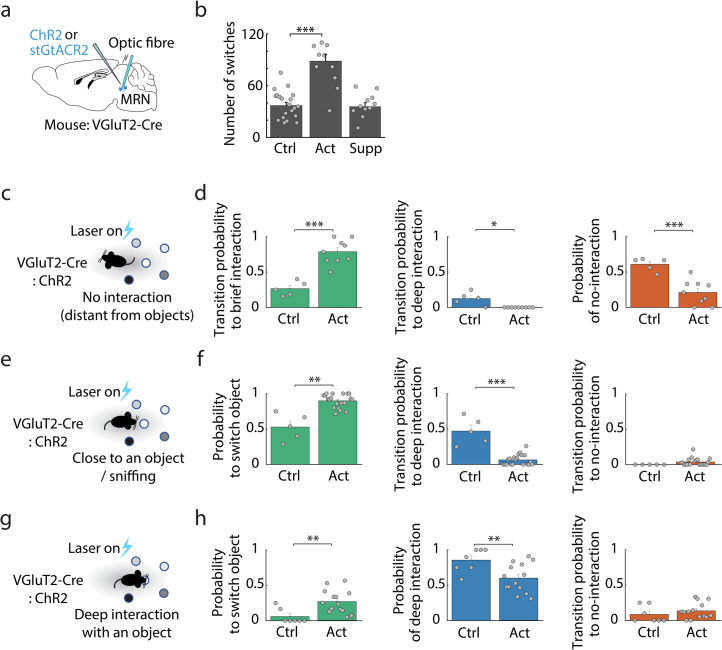
Impact of tonic and phasic optogenetic stimulation of VGluT2+ MRN neurons on object interaction. **a**, Schematic of experimental design: optogenetic activation or suppression of VGluT2+ MRN neurons, using ChR2 or stGtACR2. **b**, Number of switches between objects during the MNOI test in control mice (ctrl) and mice with activation (act) and suppression (supp) of VGluT2+ MRN neurons (ctrl vs. act: P = 3.2 × 10^−6^, ctrl vs. supp: P > 0.9999, two-sided t-test with Bonferroni multi-comparison correction). N = 20, 10 and 11 experiments from 10, 5 and 5 mice in control, VGluT2+ activation and VGluT2+ suppression groups. **c**, Schematic of the experimental design to examine the effect of phasic 2-second activation of VGluT2+ MRN neurons in the MNOI test (with 5 objects) when the animal was not interacting with an object. **d**, Transition probability from no object interaction within the 2-second stimulation window (in experiment in **c**) to brief interaction (left, tdTomato ctrl (N = 5 experiments from 5 mice) vs VGluT2+ act. (N = 9 experiments from 7 mice), P = 4.7 × 10^−5^; two-sided t-test), to deep interaction (middle, ctrl vs VGluT2+ act.: P = 0.0100; two-sided Mann-Whitney U test), and probability to remain not interacting with the objects (right, ctrl vs VGluT2+ act.: P = 4.6 × 10^−4^; two-sided t-test). **e**, Phasic 2-second activation of VGluT2+ MRN neurons in the MNOI test when the animal’s snout was close to an object. **f**, Probability within the 2-second stimulation window (in experiment in **e**) to switch to a different object (left, ctrl (N = 5 experiments from 5 mice) vs VGluT2+ act. (N = 20 experiments from 7 mice), P = 0.0012; two-sided Mann-Whitney U test), to transition to a deep interaction (middle, ctrl vs VGluT2+ act.: P = 7.4 × 10^−4^; two-sided Mann-Whitney U test), and to transition to no-interaction (right, ctrl vs VGluT2+ act.: P = 0.0602; two-sided Mann-Whitney U test). **g**, Phasic 2-second activation of VGluT2+ MRN neurons in the MNOI test during deep interaction with an object. **h**, Probability (in experiment in **g**) to switch object (left, ctrl (N = 7 experiments from 5 mice) vs VGluT2+ act. (N = 14 experiments from 7 mice), P = P = 0.0076; two-sided Mann-Whitney U test), to persist in deep interaction (middle, ctrl vs VGluT2+ act.: P = 0.0090; two-sided t-test), and to transition to no-interaction (right, ctrl vs VGluT2+ act.: P = 0.2126; two-sided Mann-Whitney U test). *: p-value < 0.05, **: p-value < 0.01, ***: p-value < 0.001. In **b**, **d**, **f** and **h** bars depict mean, error bars are standard error and circles indicate individual experiments.

Next, we tested whether VGluT2-positive MRN (MRN^VGluT2^) neurons also have a role in regulating interaction states of mice in the MNOI test. We expressed ChR2 or stGtACR2 in the MRN of VGluT2-Cre mice to optogenetically activate or deactivate MRN^VGluT2^ neurons, respectively (Fig. 2a). Activating MRN^VGluT2^ neurons in the MNOI test significantly increased the time mice spent in an exploratory state, and specifically decreased the duration of perseverative states, compared with control mice (Fig. 2b,c and Supplementary Video 3). Additionally, mice switched more frequently between different objects when MRN^VGluT2^ neurons were activated (Extended Data Fig. 6a,b), further indicating increased exploratory behaviour. Basic motor behaviour without the presence of objects was not affected during this 2-min stimulation (Extended Data Fig. 3a–e). However, activation of MRN^VGluT2^ neurons could switch a mouse’s behavioural strategy on a fast timescale, as even within a brief 2-s optogenetic activation of MRN^VGluT2^ neurons, mice were more likely to enter exploratory behaviour in the MNOI test (Extended Data Fig. 6c–h). Suppression of MRN^VGluT2^ neurons in the MNOI test did not evoke significant changes in the duration of any of the states (Fig. 2b,c), or in how often mice switched between objects (Extended Data Fig. 6a,b). Calcium signals recorded with fibre photometry (Fig. 2d) showed that activity of MRN^VGluT2^ neurons increased significantly during the exploratory state and when mice switched from one object to another, but was not different from baseline during deep interactions (Fig. 2e,f), indicating that MRN^VGluT2^ neurons are indeed involved in driving exploratory behaviour in this behavioural paradigm.

We again used the T-maze task to test whether MRN^VGluT2^ neuron activation also promotes exploratory choices in a learned task in which food-deprived mice act on previously gained knowledge. After mice learned that food reward was provided consistently only in one specific arm and not the other, MRN^VGluT2^ neurons expressing ChR2 were optogenetically activated in well-trained mice in the central arm of the T-maze (Fig. 2g). During this manipulation, mice were more likely to choose the non-rewarded arm—that is, the exploratory option (Fig. 2h)—consistent with the increase in exploratory behaviour caused by MRN^VGluT2^ neuron activation in the MNOI test (Fig. 1b,c). Moreover, calcium activity of MRN^VGluT2^ neurons selectivity increased when trained mice chose the non-rewarded arm of the T-maze (Extended Data Fig. 4f–i). However, suppression of MRN^VGluT2^ neurons did not in turn induce a bias towards perseverative choices, as mice did not choose the previously rewarded arm more often than control mice when the reward location was reversed (Fig. 2i,j). This was again consistent with the lack of effect of MRN^VGluT2^ neuron suppression on behaviour during the MNOI test (Fig. 2b,c).

These findings indicate that although activation of MRN^VGluT2^ neurons is not necessary for exploratory behaviour, it strongly promotes exploratory choices during both instinctive and learned goal-directed behaviours. Intriguingly, activation of MRN^VGAT^ neurons had also increased exploratory choices in the T-maze test (Fig. 1i,j). However, this test with two forced choices cannot distinguish whether these manipulations actively drive exploratory choices or merely prevent perseverance. To discern these possibilities, we assessed the effect of activating either MRN^VGluT2^ or MRN^VGAT^ neurons during another behavioural task—a nose poke–reward association task with distractors (Extended Data Fig. 5k). In this task, water-restricted mice learned to first insert their snout into a nose port to then receive a water reward upon licking the water port on the opposite wall. The other walls held additional nose ports that were irrelevant for the task. Optogenetic activation of MRN^VGluT2^ neurons biased mice away from the task, towards exploration of the task-irrelevant nose ports (Extended Data Fig. 5l). Activation of MRN^VGAT^ neurons, by contrast, had no effect on behaviour and did not evoke exploration of the task-irrelevant nose ports (Extended Data Fig. 5m), indicating that only activity of MRN^VGluT2^ neurons actively drives exploratory behaviour. Additionally, we confirmed this effect in the three-armed bandit task: brief optogenetic activating of MRN^VGluT2^ neurons during a period of stable exploitation of the arm with high reward probability caused mice to explore the other two arms (Extended Data Fig. 5f–j).

## MRN neurons modulate affective state

**Fig. 3:**
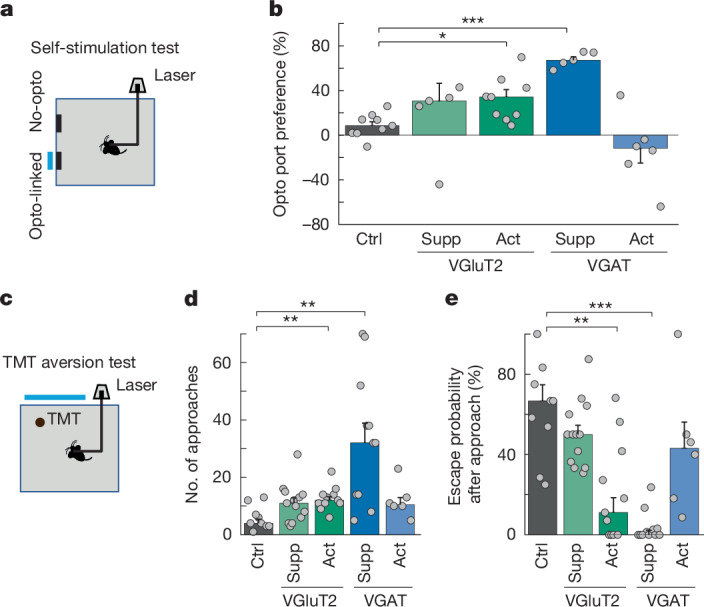
Effect of manipulation of VGAT- and VGluT2-expressing MRN neurons on valence. **a**, Schematic of the experimental design for a self-stimulation test. **b**, Preference for returning to the opto-linked nose-poke port (100 × (number of entries into the opto-linked nose-poke port − number of entries into the non-stimulation nose-poke port)/total number of entries into both nose-poke ports) in tdTomato control mice (*n* = 9) and mice with activation or suppression of VGAT^+^ (*n* = 5 and 6 mice) and VGluT2^+^ (*n* = 5 and 9 mice) MRN neurons. **c**, Schematic of the TMT aversion test. **d**, Number of approaches of the aversive TMT-coated object in control mice compared to mice with activation or suppression of VGAT^+^ and VGluT2^+^ MRN neurons. **e**, Escape probability after approaching a TMT-coated object in control mice (*n* = 9) compared with mice with VGluT2^+^ suppression (*n* = 13 mice), VGluT2^+^ activation (*n* = 11), VGAT^+^ suppression (*n* = 11) and VGAT^+^ activation (*n* = 6). *P < 0.05; **P < 0.01; ***P < 0.001. In **b**,**d**,**e**, bars indicate median, error bars are bootstrapped standard error and circles represent individual experimental sessions. See Extended Data Table 1 for statistics. Source data

**Extended Data Fig. 7:**
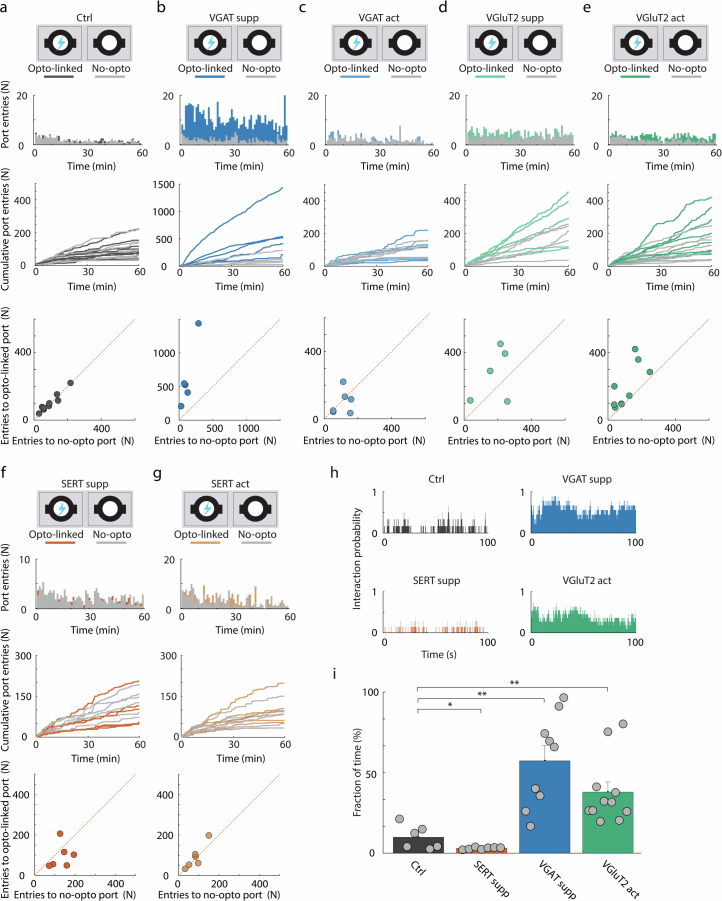
Effect of manipulation of MRN cell types on reinforcement. **a**, Top: schematic of the self-stimulation test in control mice expressing tdTomato in MRN (N = 9). Second row: number of entries (irrespective of time spent in the port) into the opto-linked (black) and non-stimulation (grey) nose-poke ports over the course of the test (averaged over mice). Third row: cumulative number of entries into the opto-linked (black) and non-stimulation (grey) nose-poke ports over the course of the test. Each line shows behaviour in an individual experimental session. Bottom: number of entries into the opto-linked port plotted against number of entries into the non-stimulation nose-poke port for individual experiments. **b**, The same as **a** but for mice with suppression of VGluT2+ MRN neurons (N = 5). **c**, The same as **a** but for mice with activation of VGluT2+ MRN neurons (N = 9). **d**, The same as **a** but for mice with suppression of VGAT+ MRN neurons (N = 5). **e**, The same as **a** but for mice with activation of VGAT+ MRN neurons (N = 6). **f**, The same as **a** but for mice with suppression of SERT+ MRN neurons (N = 6). **g**, The same as **a** but for mice with activation of SERT+ MRN neurons (N = 6). **h**, Probability of interaction with objects over the course of an MNOI test (without laser stimulation), one minute after a 2-min MNOI test with laser stimulation, averaged over mice in tdTomato control group (ctrl, N = 6 experiments from 6 mice), and in groups of mice with suppression (supp) of SERT+ and VGAT+, and activation (act) of VGluT2+ MRN neurons (N = 7, 9 and 11 experiments from 7 mice in each group). **i**, Fraction of time spent in interaction with objects in mice shown in **h**. Ctrl vs. supp. SERT: P = 0.0420, ctrl vs. supp. VGAT: P = 0.0024, ctrl vs. act. VGluT2: P = 0.0019, two-sided t-test with Bonferroni multi-comparison correction. Bars indicate median values, error bars are bootstrapped standard error and circles individual experimental sessions. *: p-value < 0.05, **: p-value < 0.01.

Changes in arousal level and valence—the affective signed value associated with a stimulus or context—are crucial for the manifestation of motivational states^30^, and are therefore likely to have an important role in regulating interactions with the environment. We thus aimed to determine to what degree changes in affective state can explain the effects of MRN^VGluT2^ and MRN^VGAT^ neuron manipulation on behavioural strategies. We used a self-stimulation test that is commonly used to assess the valence of a neural manipulation^31,32^, in which mice are presented with two nose-poke ports, only one of which triggers optogenetic self-stimulation upon entering (opto-linked port; Fig. 3a). Suppression of MRN^VGAT^ neurons led to a very strong preference to return to the opto-linked port, indicating that this manipulation is reinforcing and induces strong positive valence (Fig. 3b and Extended Data Fig. 7a,b). We also observed a reinforcing effect of this manipulation in the MNOI test (Extended Data Fig. 7h,i) The positive valence conveyed by MRN^VGAT^ neuron suppression was so dominant that it could even override strongly aversive cues. When normal mice were presented with an object coated with an innately highly aversive substance, trimethylthiazoline (TMT; a constituent of fox urine)^33^, they usually carefully approached the TMT object only a few times, often followed by a fast retreat (‘escape’; Fig. 3c–e, Supplementary Video 4 and Methods). Suppression of MRN^VGAT^ neurons markedly increased how often mice approached the TMT object, prevented escapes from it (Fig. 3d,e) and, remarkably, even elicited deep interactions with the normally highly aversive object (Supplementary Video 5), an action that is never observed in normal mice. These findings show that suppression of MRN^VGAT^ neurons induces perseverance in the current choice or goal by bestowing highly positive valence, overriding other motives.

We repeated the above tests with either activation of MRN^VGluT2^ neurons or activation of MRN^VGAT^ neurons that had elicited similar results in the T-maze test (Figs. 1i,j and 2g,h). Different to a previous study^26^, we found that activation of MRN^VGluT2^ neurons was not aversive (Extended Data Fig. 3f–h), but conveyed positive valence, as it increased the preference of mice for returning to the opto-linked port in the self-stimulation test and for returning to objects explored during stimulation in the MNOI test (Fig. 3b and Extended Data Fig. 7d,e,h,i). Moreover, the manipulation increased the number of approaches of and decreased escape probability from the aversive TMT object (Fig. 3d,e), indicating that it can even drive exploration of aversive cues. Of note, phasic activation of MRN^VGluT2^ neurons also induced increased levels of locomotion in a head-fixed preparation (Extended Data Fig. 3i,j) and increased arousal, as measured from changes in pupil size and whisking activity (Extended Data Fig. 3k,l). By contrast, activation of MRN^VGAT^ neurons did not significantly induce positive valence or increase arousal or locomotion (Fig. 3b,d,e and Extended Data Fig. 3i–n), providing further evidence that this manipulation does not induce an active state of exploration.

## SERT^+^ MRN neurons modulate engagement

**Fig. 4:**
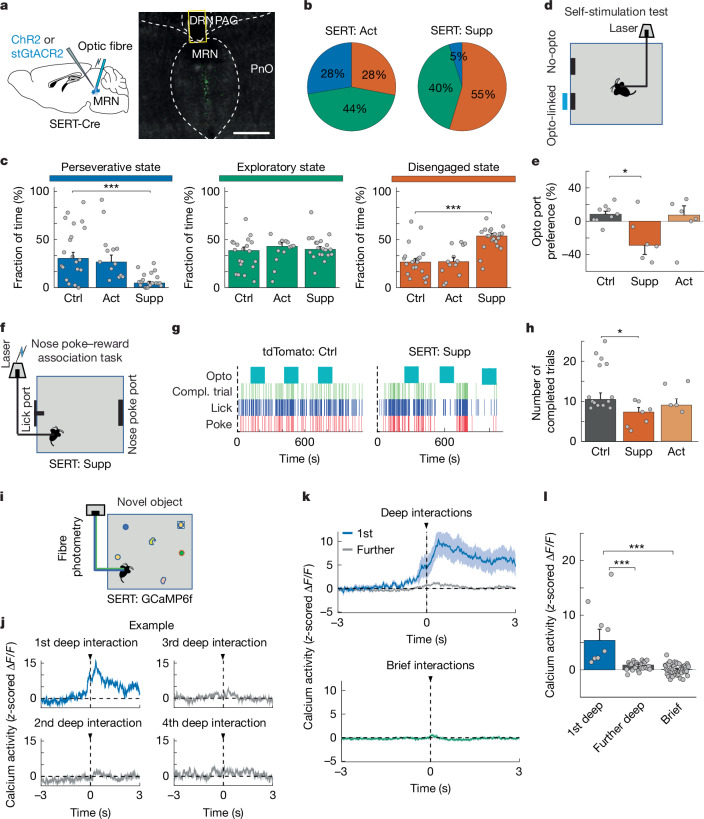
Activity of SERT-expressing MRN neurons is necessary for task engagement. **a**, Left, experimental design of optogenetic activation or suppression of serotonergic (SERT^+^) MRN neurons. Right, image of virus expression in SERT^+^ MRN neurons with optic fibre position. Scale bar, 0.5 mm. **b**,**c**, Median fraction of time spent in each interaction state during the MNOI test in mice with activation (**b**, left) or suppression (**b**, right) of SERT^+^ MRN neurons and in tdTomato control mice (**c**). *n* = 20, 13 and 20 experiments from 10, 4 and 8 mice in tdTomato, SERT+ activation and SERT+ suppression groups, respectively. **d**, Experimental design of a self-stimulation test. **e**, Preference for returning to the opto-linked nose-poke port (100 × (number of entries into the opto-linked nose-poke port − number of entries into the non-stimulation nose-poke port)/total number of entries into both nose-poke ports) in control mice (*n* = 9) and mice with activation (*n* = 6) or suppression (*n* = 6) of SERT^+^ MRN neurons. **f**, Experimental design of the nose poke–reward association task. **g**, Examples of task performance (including timing of nose pokes, licks and completed (compl.) trials) of a tdTomato control mouse and a mouse with suppression of SERT^+^ MRN neurons. Blue boxes indicate photo-stimulation time periods. **h**, Number of completed trials during photo-stimulation in tdTomato control mice and mice with suppression or activation of SERT^+^ MRN neurons. *n* = 14, 7 and 5 mice, respectively. **i**, Schematic of calcium fibre photometry recording from SERT^+^ MRN neurons in mice exposed to multiple objects. **j**, Example traces of *z*-scored calcium activity of SERT^+^ MRN neurons, aligned to the onset of object interactions, for the first (blue), second, third and fourth (grey) deep interaction with the same object. **k**, Average *z*-scored calcium activity trace (mean ± s.e.m.) during object interactions for first (top; blue; 8 trials) and following deep interactions (top; grey; 25 trials), and during brief interactions (bottom; green; 52 trials). *n* = 4 mice. **l**, Median *z*-scored calcium response of SERT^+^ MRN neurons during the first deep interaction (*n* = 8 events) compared with following deep interactions with the same object (*n* = 25 events) and brief interactions (*n* = 52 events) from 4 mice. *P < 0.05; ***P < 0.001. In **c**,**e**,**h**,**l**, bars indicate median, error bars are bootstrapped standard error and circles represent individual experimental sessions. See Extended Data Table 1 for statistics. Source data

**Extended Data Fig. 8:**
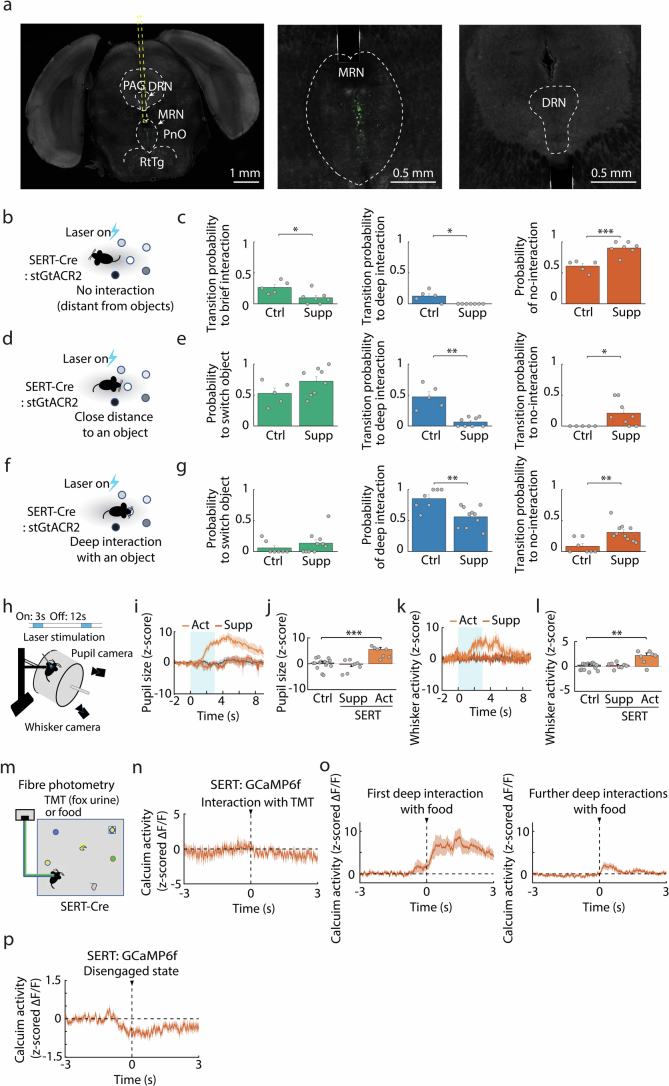
Impact of SERT+ MRN neuron phasic stimulation on engagement and arousal. **a**, Example image of virus expression (stGtACR2-fusionRed) in SERT+ neurons in the MRN with optic fibre position (left), with zoomed-in sections showing labelled neurons in MRN (middle) but not in the dorsal raphe nucleus (DRN, right). **b**, Schematic of the experimental design to examine the effect of phasic 2-second suppression of SERT+ MRN neurons in the MNOI test (with 5 objects) when the animal was not interacting with an object. **c**, Transition probability from no object interaction within the 2-second stimulation window (in the experiment in **b**) to brief interaction (left, tdTomato control mice (ctrl, N = 5 experiments from 5 mice) vs SERT+ supp. (N = 7 experiments from 7 mice), P = 0.0174; two-sided t-test), to deep interaction (middle, ctrl vs SERT+ supp.: P = 0.0202; two-sided Mann-Whitney U test), and probability to remain not interacting with objects (right, ctrl vs SERT+ supp.: P = 4.4 × 10^−4^; two-sided t-test). **d**, Phasic 2-second suppression of SERT+ MRN neurons in the MNOI test when the animal’s snout was close to an object. **e**, Transition probability within the 2-second stimulation window (in experiment in **d**) to switch to another object (left, ctrl (N = 5 experiments from 5 mice) vs SERT+ supp. (N = 8 experiments from 7 mice), P = 0.1330; two-sided t-test), to transition to a deep interaction (middle, ctrl vs SERT+ supp.: P = 0.0016; two-sided Mann-Whitney U test), and to transition to no-interaction (right, ctrl vs SERT+ supp.: P = 0.0326; two-sided Mann-Whitney U test). **f**, Phasic suppression of SERT+ MRN neurons in the MNOI test during deep interaction with an object. **g**, Probability (in experiment in **f**) to switch object within the 2-second time window (top, ctrl (N = 7 experiments from 5 mice) vs SERT+ supp. (N = 10 experiments from 7 mice), P = 0.3579; two-sided Mann-Whitney U test), to persist in deep interaction (middle, ctrl vs SERT+ supp.: P = 0.0024; two-sided t-test), and to transition to no-interaction (right, ctrl vs SERT+ supp.: P = 0.0062; two-sided Mann-Whitney U test). Bars in **c**, **e** and **g** depict mean, error bars are standard error and circles indicate individual experiments. **h**, Schematic of the experimental design to examine the effects of optogenetic manipulation of SERT+ MRN neurons on arousal level, measured using pupil size and whisker activity. **i**, Z-scored pupil size over time (mean ± s.e.m.) in control mice (grey) and mice with activation or suppression of SERT+ MRN neurons, averaged over trials and aligned to onset of optogenetic manipulation. Light blue box indicates the laser stimulation period. **j**, Median z-score of pupil size during laser stimulation from traces in **i**. N = 15, 8 and 7 mice; P > 0.9999 and P = 0.0004 for comparing control mice to activation or suppression of SERT+ neurons, respectively; two-sided t-test with Bonferroni multi-comparison correction. **k**,**l**, same as **i**,**j** but for z-scored whisker activity (summation of absolute frame-by-frame differences in pixel luminance). N = 15, 8 and 7 mice, respectively; P > 0.9999 and P = 0.0010 for comparing control mice to activation or suppression of SERT+ neurons, respectively; two-sided t-test with Bonferroni multi-comparison correction. Bars in **j** and **l** depict median, error bars are bootstrapped standard error and circles indicate individual experiments. **m**, Schematic of the fibre photometry recording during interactions with food or an aversive TMT-coated object. **n**, Average z-scored calcium trace (mean ± s.e.m., n = 4 mice) of SERT+ MRN neurons aligned to the onset of interactions with an aversive, TMT-covered object. **o**, Average z-scored calcium trace of SERT+ MRN neurons aligned to the first deep interaction (left) and further deep interactions with a food pellet (right). **p**, Average z-scored calcium trace of SERT+ MRN neurons aligned to the onset of disengaged state events (N = 75 events from 5 mice) during the MNOI test. *: p-value < 0.05, **: p-value < 0.01, ***: p-value < 0.001.

The MRN is a main source of the neuromodulator serotonin, which is commonly implicated in behavioural flexibility and perseverative behaviour^13,18–21,34^. We therefore tested whether MRN serotonergic (MRN^SERT^) neurons also have a role in balancing exploratory and perseverative choices. We expressed inhibitory opsin stGtACR2 or excitatory opsin ChR2 specifically in MRN^SERT^ neurons of SERT-Cre mice (Extended Data Figs. 1d–f and 8a), to suppress or activate these neurons, respectively, during the MNOI test, and to quantify the effect of MRN^SERT^ neuron manipulation on the duration of perseverative, exploratory and disengaged states (Fig. 4a). Suppression of MRN^SERT^ neurons resulted in a large increase in the time mice spent in a disengaged state and decreased the duration of perseverative states compared with control mice (Fig. 4b,c). This effect was even apparent on a much faster timescale, as mice were more likely to disengage from objects during a brief 2-s optogenetic suppression (Extended Data Fig. 8b–g). By contrast, optogenetic activation of these neurons had no effect on how long mice spent in each of the states (Fig. 4b,c).

In the self-stimulation test, suppression of MRN^SERT^ neurons decreased the preference of the mice for the opto-linked port, compared with control mice (Fig. 4d,e), indicating that it conveys negative valence. This negatively reinforcing effect was also apparent in the MNOI test after MRN^SERT^ neuron suppression (Extended Data Fig. 7h,i). However, MRN^SERT^ neuron suppression had no effect on measures of arousal or basic motor behaviour (Extended Data Figs. 3a–e and 8h–l). Next, we examined whether suppression of MRN^SERT^ neurons also causes disengagement during learned goal-directed behaviours. To this end, we optogenetically suppressed these neurons during a nose poke–reward association task, in which water-restricted mice had learned to first poke into a nose port to subsequently receive a water reward upon licking a water port on the opposite wall (in this case without distractor ports; Fig. 4f). In this task, the frequency of completed trials indicates the level of engagement of the mouse. When suppressing MRN^SERT^ neurons, mice completed significantly fewer trials compared with control mice (Fig. 4g,h and Supplementary Video 6), confirming that activity of MRN^SERT^ neurons is necessary for task engagement. By contrast, activation of MRN^SERT^ neurons had no effect on engagement levels (Fig. 4g,h).

We recorded calcium signals using fibre photometry after expression of Cre-dependent GCaMP6f in MRN of SERT-Cre mice to test how activity of MRN^SERT^ neurons is modulated during object interactions (Fig. 4i). Activity of MRN^SERT^ neurons decreased when mice disengaged from objects (Extended Data Fig. 8p), consistent with the effect of optogenetic suppression on behaviour. By contrast, MRN^SERT^ neuron activity was markedly increased during the first deep interactions of mice with a novel object. Notably, further deep or brief interactions with the same item did not cause a calcium response (Fig. 4j–l), suggesting that increased MRN^SERT^ neuron activity indicates novelty or salience of an item, similar to DRN serotonin neurons^35^. We observed a similar pattern of activation when mice interacted with food items instead of novel objects (Extended Data Fig. 8m,o). However, interactions with novel objects of negative valence—objects coated in aversive TMT—did not evoke changes in the average MRN^SERT^ neuron calcium activity (Extended Data Fig. 8n). However, this method cannot exclude that some individual MRN^SERT^ neurons may respond to such stimuli, as is the case for DRN serotonin neurons^35^.

Our results show that these MRN^SERT^ signals in response to positive salience are necessary for task engagement and sustained interaction with resources. Although MRN^SERT^ neuron activation on its own is not sufficient to induce task engagement or sustained interactions, suppression of MRN^SERT^ neurons causes disengagement, potentially by decreasing salience and endowing current choices with negative valence.

Together, these findings indicate that MRN^VGAT^ neuron suppression and MRN^VGluT2^ neuron activation cause perseverative and exploratory choices, respectively. By contrast, MRN^SERT^ activity does not regulate explore–exploit decisions, but is necessary for engagement with environmental resources and the continuing pursuit of goals.

## LHb input to MRN promotes disengagement

**Fig. 5:**
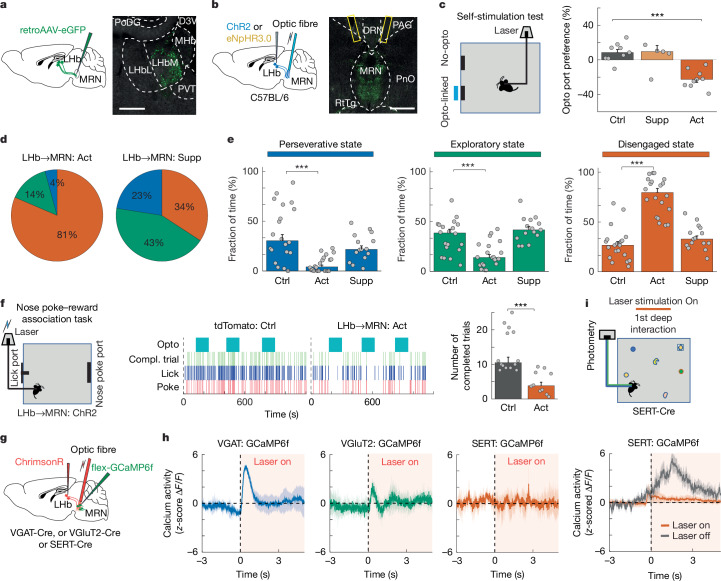
LHb input to MRN drives disengagement. **a**, Retrograde tracing from MRN using retroAAV (left), and image of retrogradely labelled neurons in LHb (right). Scale bar, 0.25 mm. **b**, Schematic of optogenetic activation or suppression of LHb input to MRN (left), and image of ChR2-expressing LHb axons in the MRN with optic fibre positions (right). Scale bar, 0.5 mm. **c**, Schematic of the self-stimulation test (left), and preference for returning to the opto-linked nose-poke port (calculated as in Fig. 4e) in control mice compared with mice with suppression or activation of LHb input to the MRN (right; *n* = 9, 5 and 9 mice, respectively). **d**,**e**, Median fraction of time spent in the three interaction states during the MNOI test in mice with activation (**d**, left) and suppression (**d**, right) of LHb input to the MRN and in control mice (**e**). *n* = 20, 20 and 15 experiments from 10, 9 and 5 mice in control, activation and suppression groups, respectively. **f**, Schematic of nose-poke reward association task with activation of LHb input to the MRN (left) and examples of task performance (including timing of nose pokes, licks and completed (compl.) trials) of a control mouse and a mouse with activation of LHb input to the MRN. Light blue boxes indicate laser stimulation periods. Right, average number of completed trials during laser stimulation in tdTomato mice and mice with activation of LHb input in the MRN (*n* = 14 and 10 mice, respectively). **g**, Experimental design for recording calcium signals in MRN cell types using fibre photometry, while optogenetically activating LHb input to the MRN. **h**, *z*-Scored calcium traces (mean ± s.e.m.) aligned to laser onset, showing the effect of optogenetic activation of LHb input on the activity of VGAT^+^ (left; *n* = 4 mice), VGluT2^+^ (middle; *n* = 5 mice) and SERT+ (right; *n* = 4 mice) neurons in MRN. **i**, Top, schematic of experimental design to obtain calcium recordings from SERT^+^ MRN neurons, while activating LHb input to the MRN during the first deep interaction with objects. Bottom, *z*-scored calcium activity (mean ± s.e.m.) aligned to onset of first deep object interaction with and without optogenetic activation of LHb input to the MRN. *n* = 10 (laser off) versus 7 (laser on) events from 3 mice. ***P < 0.001. In **c**,**e**,**f**, bars indicate median values, error bars are bootstrapped standard error and circles represent individual experimental sessions. See Extended Data Table 1 for statistics. Source data

**Fig. 6:**
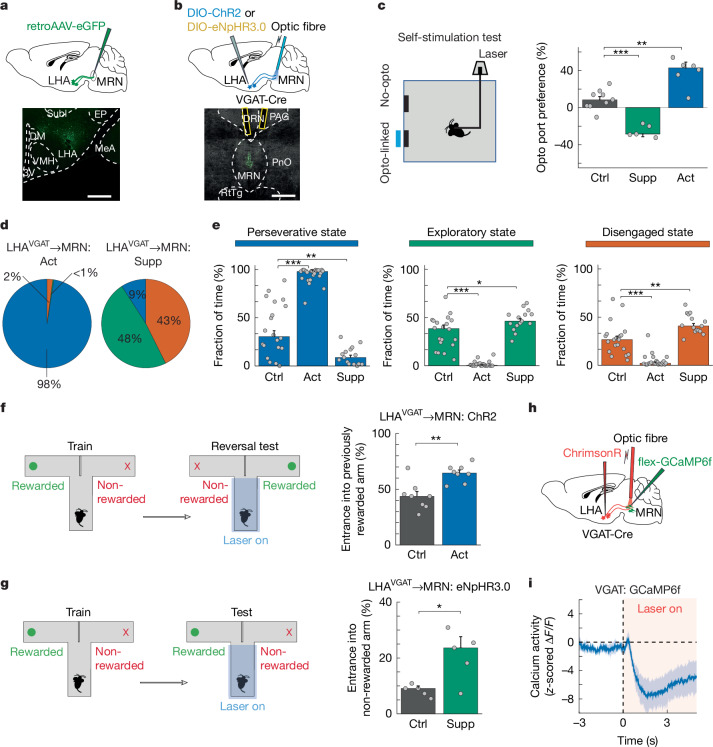
LHA input to MRN bidirectionally controls perseverance. **a**, Retrograde tracing from MRN using retroAAV (top), and image of retrogradely labelled neurons in LHA (bottom). 3V, 3rd ventricle; DM, dorsomedial hypothalamus; EP, entropeduncular nucleus; MeA, medial amygdala; Subl, subincertal nucleus; VMH, ventromedial hypothalamus. Scale bar, 0.5 mm. **b**, Schematic of optogenetic activation or suppression of LHA VGAT^+^ input to MRN (top) and image of LHA axons in MRN with optic fibre positions (bottom). Scale bar, 0.5 mm. **c**, Schematic of the self-stimulation test (left), and preference for returning to the opto-linked nose-poke port (calculated as in Fig. 4e) in control mice (*n* = 9) and mice with suppression or activation of LHA VGAT^+^ input to the MRN (right; *n* = 5 and 6 mice, respectively). **d**,**e**, Median fraction of time spent in the interaction states during the MNOI test in mice with activation (**d**, left) and suppression (**d**, right) of the LHA VGAT^+^ input to the MRN and in control mice (**e**). *n* = 20, 23 and 15 experiments from 10, 9 and 8 mice in control mice, activation and suppression groups. **f**, Left, experimental design to quantify levels of perseverance during a reversal test in the T-maze. Right, percentage of trials with entrance into the previously rewarded (but now non-rewarded) arm in control mice (*n* = 8) and mice with activation of LHA VGAT^+^ input to the MRN (8 mice). **g**, Left, experimental design to quantify levels of exploration during a T-maze test. Right, percentage of trials with entrance into the non-rewarded arm in control mice (*n* = 5) and mice with suppression of LHA VGAT^+^ input to the MRN (5 mice). **h**, Experimental design for recording calcium signals in MRN VGAT^+^ neurons while optogenetically activating LHA VGAT^+^ input to the MRN. **i**, *z*-scored calcium traces (mean ± s.e.m.) aligned to laser onset, showing the effect of optogenetic activation of LHA VGAT^+^ input on the activity of the VGAT^+^ MRN neurons (*n* = 5 mice). *P < 0.05; **P < 0.01; ***P < 0.001. In **c**,**e**–**g**, bars indicate median values, error bars are bootstrapped standard error and circles represent individual experimental sessions. See Extended Data Table 1 for statistics. Source data

**Extended Data Fig. 9:**
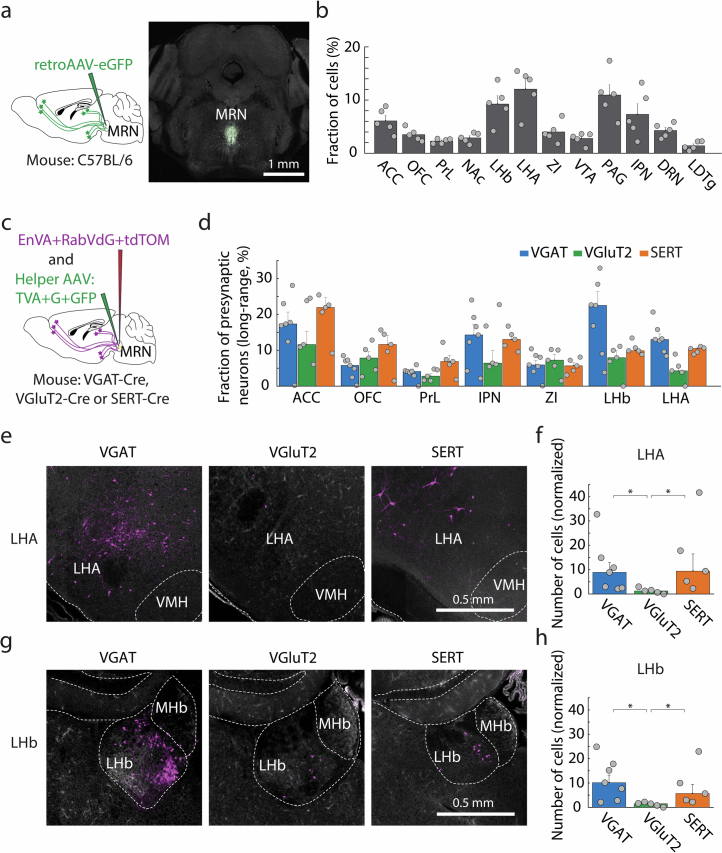
Inputs to MRN and specific MRN cell types. **a**, Schematic of experimental design to express eGFP in MRN-projecting neurons, using a retrograde AAV (left) and example image of the virus expression in MRN (right). **b**, Fraction of MRN-projecting cells out of all eGFP-expressing cells in the brain in the brain areas with highest density of labelled neurons (N = 5 mice). ACC: Anterior cingulate cortex, OFC: Orbitofrontal cortex, PrL: Prelimbic cortex, NAc: Nucleus accumbens, LHb: Lateral habenula, LHA: Lateral hypothalamic area, ZI: Zona incerta, VTA: ventrolateral tegmental area, PAG: Periaqueductal gray, IPN: Interpeduncular nucleus, DRN: Dorsal raphe nucleus, LDTg: Laterodorsal tegmental nucleus. **c**, Schematic of experimental design to label neurons presynaptic of VGAT+, VGluT2+, and SERT+ neurons in MRN, using a mono-transsynaptic rabies virus approach. **d**, Fraction of neurons innervating VGAT+ MRN neurons (blue), VGluT2+ MRN neurons (green), and SERT + MRN neurons (orange) out of the total number of presynaptic neurons from long-range projections (excluding areas close to the MRN, such as DRN and PAG; N = 7, 5 and 5 VGAT-Cre, VGluT2-Cre, and SERT-Cre mice). ACC: Anterior cingulate cortex, OFC: Orbitofrontal cortex, PrL: Prelimbic cortex, LHb: Lateral habenula, LHA: Lateral hypothalamic area, IPN: Interpeduncular nucleus, ZI: Zona incerta. **e**, Example image of LHA neurons innervating VGAT+ MRN neurons (left), VGluT2+ MRN neurons (middle), and SERT+ MRN neurons (right). **f**, Number of VGAT+ MRN-innervating, VGluT2+ MRN-innervating, and SERT+ MRN-innervating neurons in LHA, normalized by the total number of starter cells in MRN (N = 7, 5 and 5 VGAT-Cre, VGluT2-Cre, and SERT-Cre mice). VGAT vs VGluT2: P = 0.0379, VGAT vs SERT: P > 0.9999, VGluT2 vs SERT: P = 0.0476; two-sided Mann-Whitney U test with Bonferroni multi-comparison correction. Note that this approach cannot distinguish between GABAergic and glutamatergic presynaptic neurons in the LHA. **g**, Example image of LHb neurons innervating VGAT+ MRN neurons (left), VGluT2+ MRN neurons (middle), and SERT+ MRN neurons (right). Scale bar indicates 0.5 mm. **h**, Number of VGAT+ MRN-innervating, VGluT2+ MRN-innervating, and SERT + MRN-innervating neurons in LHb, normalized by the total number of the starter cells in MRN (N = 7, 5 and 5 VGAT-Cre, VGluT2-Cre, and SERT-Cre mice). VGAT vs VGluT2: P = 0.0152, VGAT vs SERT: P > 0.9999, VGluT2 vs SERT: P = 0.0476; two-sided Mann-Whitney U test with Bonferroni multi-comparison correction. *: p-value < 0.05. Bars in **b**, **d**, **f** and **h** depict median, error bars are bootstrapped standard error and circles indicate individual experiments.

Next, we aimed to identify brain areas upstream of MRN that convey information that is relevant for the generation of perseverative, exploratory or disengaged states. Using retrograde virus tracing, we identified the lateral hypothalamic area (LHA) and lateral habenula (LHb) as two major inputs to the MRN (Figs. 5a and 6a and Extended Data Fig. 9a,b). Because LHb is thought to be associated with negative affective state and depressive-like behaviours^36,37^, we hypothesized that LHb may convey negative valence signals to MRN, which is important for regulating engagement levels.

**Extended Data Fig. 10:**
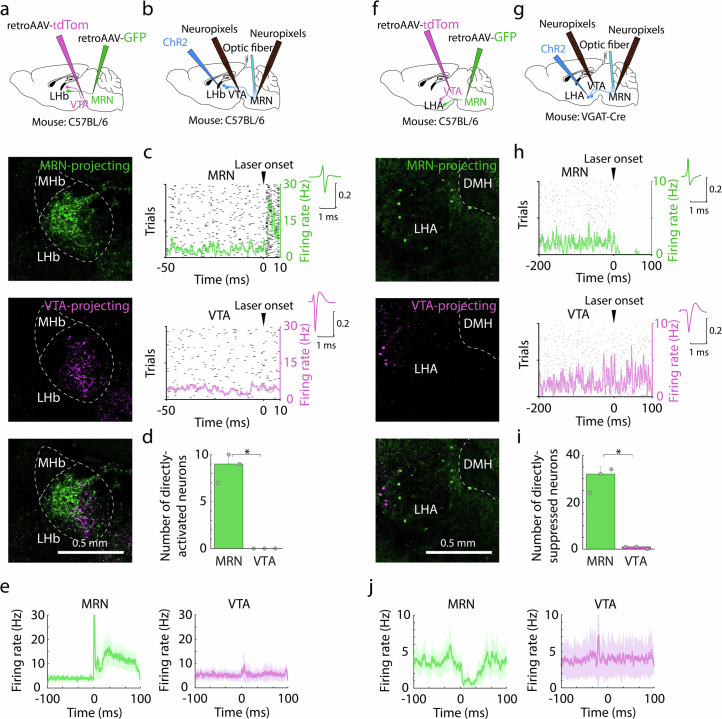
Activation of MRN inputs from LHA and LHb has little direct impact on VTA. **a**, Top, Schematic of experimental design to label MRN-projecting and ventral tegmental area (VTA)-projecting neurons, with GFP and tdTomato, respectively. Bottom, Example of MRN-projecting (green) and VTA-projecting (magenta) neurons in LHb, and their overlap. **b**, Schematic of experimental design to record the effect of activation of LHb input to MRN on the activity of MRN and VTA neurons, using high-density multi-channel Neuropixels probes. **c**, Top, Example raster plot (each row is one trial, each dot is one spike) of activity of one example MRN single-unit over the laser stimulation trials, aligned to the onset of laser stimulation. The firing rate averaged over trials is shown in green. The right green y-axis indicates the firing rate in Hz. The right-top corner shows the spike shape. Bottom, same as top but for a VTA single-unit. The firing rate averaged over trials is shown in magenta. **d**, Number of MRN and VTA neurons (median ± bootstrapped standard error) activated within 10 ms from the onset of laser stimulation of LHb input to MRN (*N* = 3 mice, *P* = 0.0102, two-sided paired t-test). **e**, Average firing rate of MRN (left) and VTA (right) single-units, while activating LHb axons in MRN, aligned to laser onset (mean ± s.e.m.). Firing rate comparison 50 ms before vs after laser onset in MRN: *P* = 7.1 × 10^−12^; and VTA: *P* = 0.98; two-sided Wilcoxon signed-rank test. **f**, Same as **a**, but for MRN-projecting (green) and VTA-projecting (magenta) neurons in LHA, and their overlap. **g**, Schematic of experimental design to record the effect of activation of LHA GABAergic input to MRN on the activity of MRN and VTA neurons, using high-density multi-channel Neuropixels probes. **h**, Same as **c**, but for optogenetic activation of GABAergic LHA axons in MRN. **i**, Number of MRN and VTA neurons (median ± bootstrapped standard error) suppressed within 100 ms from the onset of laser stimulation of LHA GABAergic input to MRN (*N* = 3 mice, *P* = 0.0123, two-sided paired t-test). **j**, Average firing rate of single units in MRN (left) and VTA (right), while activating LHA GABAergic axons in MRN, aligned to laser onset (mean ± s.e.m.). Firing rate comparison 50 ms before vs after laser onset in MRN: *P* = 2.5 × 10^−8^, and VTA: *P* = 0.15; two-sided Wilcoxon signed-rank test. *: p-value < 0.05, ***: p-value < 0.001.

**Extended Data Fig. 11:**
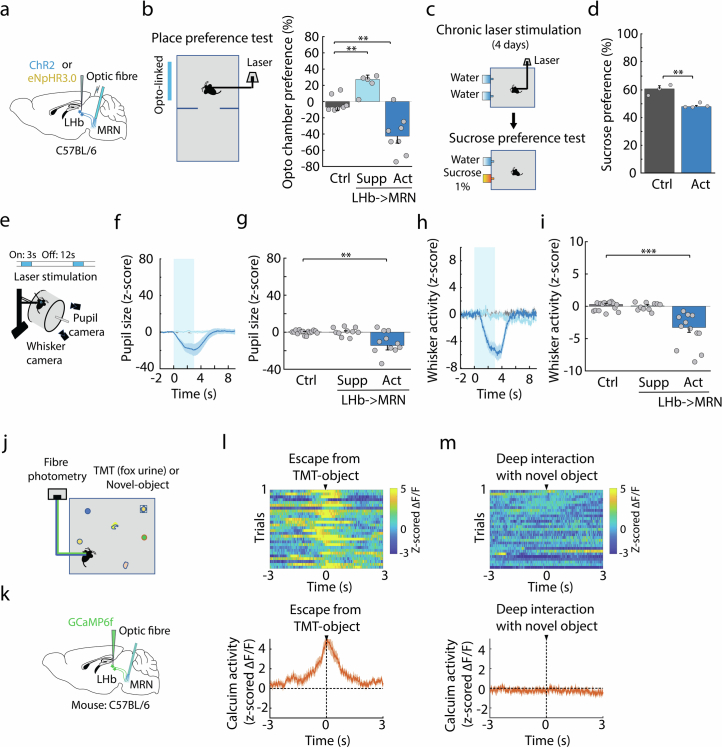
Impact of manipulation of LHb input to MRN on valence and arousal and fibre photometry calcium recordings from these projections. **a**, Schematic of experimental design for optogenetic suppression or activation of LHb input to MRN. **b**, Left: schematic of the real-time place preference test. Right: preference for the opto-linked chamber (100 × (duration of time spent in the opto-linked chamber - duration of time spent in the non-stimulation chamber) / total time) in control mice and mice with activation or suppression of LHb input to MRN (N = 7, 5 and 8 mice, respectively; P = 0.0053 and P = 0.0034 for comparing control mice to activation or suppression of LHb input to MRN, respectively; two-sided t-test with Bonferroni multi-comparison correction). **c**, Schematic of the experimental design: chronic laser activation (4 days, 24 h per day) followed by the sucrose preference test without laser stimulation on the 5th day. **d**, Sucrose preference in control mice and mice after repeated optogenetic activation of LHb input to MRN (N = 3 and 4 mice, respectively, P = 0.0027, two-sided t-test). **e**, Experimental design to examine the effects optogenetic manipulation of LHb input to MRN on arousal level, measured using pupil size and whisker activity. **f**, Z-scored pupil size over time (mean ± s.e.m.) in control mice and mice with suppression or activation of LHb input to MRN, averaged over laser stimulation trials and aligned to laser onset. The light blue box indicates the laser stimulation period. **g**, Median z-scored pupil size during the laser stimulation period from traces in **f**. N = 15, 10 and 12 mice; P > 0.9999 and P = 0.0037 for comparing control mice to suppression or activation of LHb input to MRN, respectively; two-sided t-test with Bonferroni multi-comparison correction. **h**,**i**, same as **f**,**g** but for z-scored whisker activity (summation of absolute frame-by-frame differences in pixel luminance). N = 15, 10 and 12 mice; P > 0.9999 and P = 2.4 × 10^−5^ for comparing control mice to suppression or activation of LHb input to MRN, respectively; two-sided t-test with Bonferroni multi-comparison correction. In **b**, **d**, **g** and **i** bars depict median, error bars are bootstrapped standard error and circles indicate individual experiments. **j**, Schematic of calcium fibre photometry recording from LHb input to MRN in mice exposed to either novel objects or aversive TMT-coated objects. **k**, Schematic of the experimental design to express GCaMP6f in LHb neurons and implant a fibre to image LHb input to MRN. **l**, Left, Heatmap shows z-scored calcium activity of LHb input to MRN (top) and their average z-scored calcium activity trace (mean ± s.e.m.) across mice (bottom, N = 4 mice, activity during baseline vs during escape: P = 2.9 × 10^−5^; two-sided Wilcoxon signed rank test) aligned to time of fast retreat (escape) after an approach of a TMT-coated object. **m**, Same as **l**, but calcium activity (mean ± s.e.m.) aligned to start of a deep interaction with a novel object (N = 3 mice, activity during baseline vs during deep interactions: P = 0.7084; two-sided Wilcoxon signed rank test). **: p-value < 0.01, ***: p-value < 0.001.

We tested this hypothesis by injecting adeno-associated viruses (AAVs) for expression of optogenetic constructs in LHb, and we either specifically suppressed this axonal pathway using inhibitory opsin eNpHR3.0 for axonal silencing, or optogenetically activated ChR2-expressing LHb axons in MRN (Fig. 5b) during the different behavioural paradigms introduced above. Using electrophysiological Neuropixels recordings in MRN and ventral tegmental area (VTA), we confirmed the specificity of this manipulation: activating ChR2-expressing LHb axons in MRN indeed strongly affected spiking activity in MRN but not in VTA (Extended Data Fig. 10a–e). In the self-stimulation test and a real-time place preference test, activation of LHb→MRN input significantly decreased preference of mice for returning to the opto-linked port and for remaining in the opto-linked chamber (Fig. 5c and Extended Data Fig. 11a,b), whereas silencing the LHb→MRN pathway increased preference for the opto-linked chamber (Extended Data Fig. 11b). Furthermore, chronic activation of LHb→MRN input resulted in anhedonia, as evidenced by a decrease in sucrose preference in a sucrose preference test (Extended Data Fig. 11c,d). Signals from LHb thus convey strong negative valence to MRN, consistent with previous work that linked increased activity within LHb with negative valence, disengagement and depression-like behaviour^36,37^. Activation of LHb→MRN input also decreased arousal levels (Extended Data Fig. 11e–i). We therefore tested whether LHb→MRN input could also regulate engagement of mice in different tasks. Whereas suppression of LHb→MRN input did not change the duration of interaction states in the MNOI test, activation of this pathway significantly increased the time mice spent in a disengaged state (Fig. 5d,e). LHb→MRN input activation had a similar effect on a learned goal-directed behaviour, as it also decreased engagement of mice in the nose-poke reward test (Fig. 5f and Supplementary Video 7). Fibre photometry recordings from GCaMP-expressing LHb axons in MRN showed no change in calcium activity during interactions with novel objects, but increased activity when mice encountered and escaped from aversive TMT-covered objects (Extended Data Fig. 11j–m), consistent with the negative valence conveyed by activating these projections.

Activation of the LHb projection to MRN has a similar effect on behaviour as MRN^SERT^ neuron suppression (Fig. 4b,c), even though LHb input to MRN is mostly glutamatergic^14,26^. We thus tested whether LHb activation had an excitatory or net inhibitory effect on the activity of serotonergic neurons in MRN using fibre photometry of calcium signals in MRN^SERT^ neurons combined with optogenetic activation of LHb (Fig. 5g). Indeed, LHb activation did not excite MRN^SERT^ neurons (Fig. 5h, right), but strongly suppressed their responses to novel objects (Fig. 5i). This inhibitory effect is likely to be mediated by a subset of GABAergic neurons in MRN, since MRN^VGAT^, but not MRN^VGluT2^ neurons, were on average excited by LHb activation (Fig. 5h, left and middle). In line with these functional effects, anatomical mono-synaptic rabies tracing showed particularly prominent input from LHb to MRN^VGAT^ neurons (Extended Data Fig. 9c,d,g,h). Input from LHb to MRN can thus negatively regulate engagement levels through inhibition of MRN^SERT^ neurons.

## LHA input to MRN regulates perseverance

**Extended Data Fig. 12:**
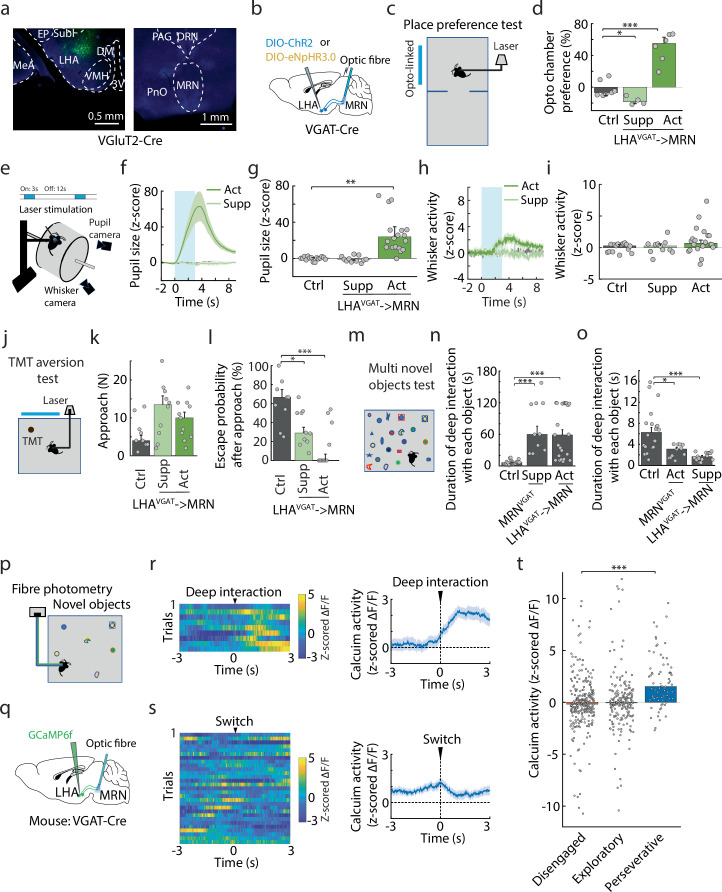
Impact of manipulation of LHA VGAT+ input to MRN on valence, arousal and deep object interactions, and fibre photometry calcium recordings from these projections. **a**, Example image of virus expression in VGluT2+ neurons in lateral hypothalamic area (LHA,in green; left) of VgluT2-Cre mice, showing absence of axon terminals in MRN (right). 3 V: 3^rd^ ventricle, DM: dorsomedial hypothalamus, EP: entropeduncular nucleus, LHA: lateral hypothalamic area, MeA: medial amygdala, Subl: subincertal nucleus, VMH: ventromedial hypothalamus. DRN: dorsal raphe nucleus, MRN: median raphe nucleus, PAG: periaqueductal gray, PnO: pontine reticular formation. **b**, Schematic of experimental design for optogenetic suppression or activation of LHA VGAT+ input to MRN. **c**, Schematic of the real-time place preference test. **d**, Preference for the opto-linked chamber (100 × (duration of time spent in the opto-linked chamber - duration of time spent in the non-stimulation chamber) / total time) in control mice and mice with activation or suppression of LHA VGAT+ input to MRN (N = 7, 4 and 6 mice, respectively; P = 0.04865 and P = 0.0001 for comparing control mice to activation or suppression of LHA VGAT+ input to MRN, respectively; two-sided t-test with Bonferroni multi-comparison correction). **e**, Experimental design to examine the effects of optogenetic manipulation of LHA VGAT + MRN input on arousal level, measured using pupil size and whisker activity. **f**, Z-scored pupil size over time (mean ± s.e.m.) in control mice and mice with suppression or activation of LHA VGAT+ input to MRN, averaged over laser stimulation trials and aligned to laser onset. The light blue bar indicates the laser stimulation period. **g**, Median z-scored pupil size during the laser stimulation period from traces in **f**. N = 15, 11 and 20 mice; P > 0.9999 and P = 0.0044, for comparing control mice to suppression or activation of LHA input, respectively; two-sided t-test with Bonferroni multi-comparison correction. **h**,**i**, same as **f**,**g** but for z-scored whisker activity (summation of absolute frame-by-frame differences in pixel luminance). N = 15, 11 and 20 mice; P > 0.9999 and P = 0.1352 for comparing control mice to suppression or activation of LHA input, respectively; two-sided t-test with Bonferroni multi-comparison correction. **j**, Schematic of the TMT aversion test. **k**, Number of approaches of the aversive TMT-covered object in control mice and mice with suppression or activation of LHA VGAT+ input to MRN. N = 9, 10 and 11 mice, respectively; P = 0.0760 and P = 0.2107 for comparing control mice to suppression or activation of LHA input, respectively; two-sided t-test with Bonferroni multi-comparison correction. **l**, Escape probability after approaching the TMT-covered object for mice shown in **k**. P = 0.0258 and P = 0.0005 for comparing control mice to suppression or activation of LHA input, respectively; two-sided t-test with Bonferroni multi-comparison correction. **m**, Schematic of the MNOI test. **n**, Duration of deep interactions with each object during the MNOI test in control mice (ctrl), mice with suppression of VGAT + MRN neurons (supp. vgat) and mice with activation of LHA VGAT+ input to the MRN (act. lha). Ctrl vs. supp. vgat: P = 1.6 × 10^−6^, ctrl vs. act. lha: P = 7.6 × 10^−6^, two-sided t-test with Bonferroni multi-comparison correction. N = 20, 10 and 23 experiments from 10, 5 and 9 mice in ctrl, supp. vgat and act. lha groups. **o**, Duration of deep interactions with each object in the MNOI test in control mice (ctrl), mice with activation of VGAT + MRN neurons (act. VGAT) and mice with suppression of LHA VGAT+ input to the MRN (supp. LHA), Ctrl vs. act. VGAT: P = 0.0102, ctrl vs. supp. LHA: P = 0.0001, two-sided t-test with Bonferroni multi-comparison correction. N = 20, 11 and 15 experiments from 10, 6 and 8 mice in ctrl, act. VGAT and supp. LHA groups. **p**, Schematic of calcium fibre photometry recording from GABAergic LHA input to MRN in mice exposed to multiple novel objects. **q**, Schematic of the experimental design to express GCaMP6f in VGAT + LHA neurons and implant a fibre to image LHA input to MRN. **r**, Heatmap of individual z-scored calcium activity traces of VGAT + LHA input to MRN of an example mouse (left) and average z-scored calcium activity trace (mean ± s.e.m.) across mice (N = 5 mice, activity during baseline vs during deep interactions: P = 5.4 × 10^−8^; two-sided Wilcoxon signed rank test) (right) during object interactions aligned to the onset of deep interaction with an object. **s**, Heatmap of z-scored calcium activity traces of VGAT + LHA input to MRN of the same mouse as **r** (left) and average z-scored calcium activity trace (mean ± s.e.m.) across mice (before-interaction baseline vs activity after switching object: P = 0.0672; two-sided Mann-Whittney U test) (right) during object interactions aligned to the time of switching between objects. **t**, Median z-scored calcium activity of VGAT + LHA input to MRN during disengaged states (N = 261 events from 5 mice), exploratory states (N = 195 events, disengaged vs exploratory P > 0.9999) and perseverative states (N = 71 events, disengaged vs perseverative: P = 0.0002), two-sided nested ANOVA with Bonferroni multi-comparison correction. *: p-value < 0.05, **: p-value < 0.01, ***: p-value < 0.001. In panels **d**, **g**, **i**, **k**, **l**, **n**, **o** and **t** bars depict median, error bars are bootstrapped standard error and circles indicate individual experiments.

LHA provides another main input to MRN and has been shown to be important for motivational behaviours^38,39^ (Fig. 6a and Extended Data Fig. 9a,b). We therefore speculated that this input may carry positive valence signals important for sustaining goal-directed behaviour. LHA→MRN input is predominantly GABAergic (Fig. 6b and Extended Data Fig. 12a), enabling us to manipulate this projection using injection of Cre-dependent ChR2 or eNpHR3.0 into the LHA of VGAT-Cre mice (Fig. 6b). Activating ChR2-expressing GABAergic LHA axons in MRN strongly suppressed activity in MRN, but not in VTA, confirming the specificity of this approach (Extended Data Fig. 10f–j). Activating GABAergic LHA^VGAT^ axons in MRN significantly increased preference of mice for returning to the opto-linked port in the self-stimulation test and for the opto-linked chamber in the real-time place preference test^26,32^, whereas selectively silencing this pathway had the opposite effect (Fig. 6c and Extended Data Fig. 12c,d). Moreover, activation of LHA^VGAT^→MRN input also increased arousal levels (Extended Data Fig. 12e–i), and could override the strong aversion of mice to TMT-coated objects, inducing deep interactions with these aversive objects (Extended Data Fig. 12j–l and Supplementary Video 8), similar to the effects of suppressing MRN^VGAT^ neurons. Thus, GABAergic LHA input to MRN conveys strong positive valence, whereas the absence of this input may impose negative valence on the current goal of the mice.

We next tested whether this projection pathway could also influence behavioural strategies during interactions with different resources. Indeed, in the MNOI test, activation of LHA^VGAT^→MRN input strongly increased the duration of perseverative states, in which mice showed sustained interactions with one or few objects, and significantly decreased the duration of both exploratory and disengaged states, compared with control mice (Fig. 6d,e and Supplementary Video 9). Moreover, this manipulation increased the time mice spent with each object (Extended Data Fig. 12m,n). Moreover, fibre photometry recordings of LHA^VGAT^ axons in MRN showed increased calcium activity during deep interactions with objects and in general during the perseverative state in the MNOI test (Extended Data Fig. 12p–t). These findings were corroborated in the T-maze test: after reversal of the reward location, food-restricted mice that had previously been trained to expect a food reward in the now unrewarded T-maze arm continued to prefer this unrewarded arm when LHA^VGAT^→MRN input was activated in the central arm of the T-maze, demonstrating perseverance in familiar, previously optimal choices (Fig. 6f). Notably, these effects closely resembled the increase in perseverative behaviour induced by suppression of MRN^VGAT^ neurons (Fig. 1d,e,l).

By contrast, inactivating LHA^VGAT^ axons in MRN in the MNOI test decreased the duration of perseverative states, while both exploratory and disengaged states were slightly prolonged (Fig. 6d,e). Notably, this manipulation also strongly decreased the time mice spent with individual objects, preventing sustained interactions (Extended Data Fig. 12m,o and Supplementary Video 10). Accordingly, silencing of LHA^VGAT^ axons in MRN decreased the fraction of perseverative choices in the T-maze test, causing mice to choose the unrewarded arm more often, even though they were trained to expect reward in the other arm (Fig. 6g). Given that LHA^VGAT^ axon silencing did not change arousal levels (Extended Data Fig. 12e–i) and suppressed perseverative behaviour in the MNOI test rather than specifically increasing the time mice spend in an exploratory state, this manipulation is likely to prevent sustained engagement in a specific action or goal, rather than driving exploratory choices. Of note, these effects resembled the results of MRN^VGAT^ neuron activation, which had also disrupted sustained object interactions in the MNOI test (Extended Data Fig. 12m–o). We thus tested whether the influence of LHA^VGAT^ projections in MRN on perseverative behaviour could be mediated through their inhibitory influence on MRN^VGAT^ neurons. Indeed, fibre photometry recordings of calcium activity in MRN^VGAT^ neurons during optogenetic LHA^VGAT^ neuron manipulation confirmed that activation of LHA^VGAT^→MRN input inhibited MRN^VGAT^ neurons (Fig. 6h,i), explaining the similar effect of these neural manipulations on behaviour.

**Extended Data Fig. 13:**
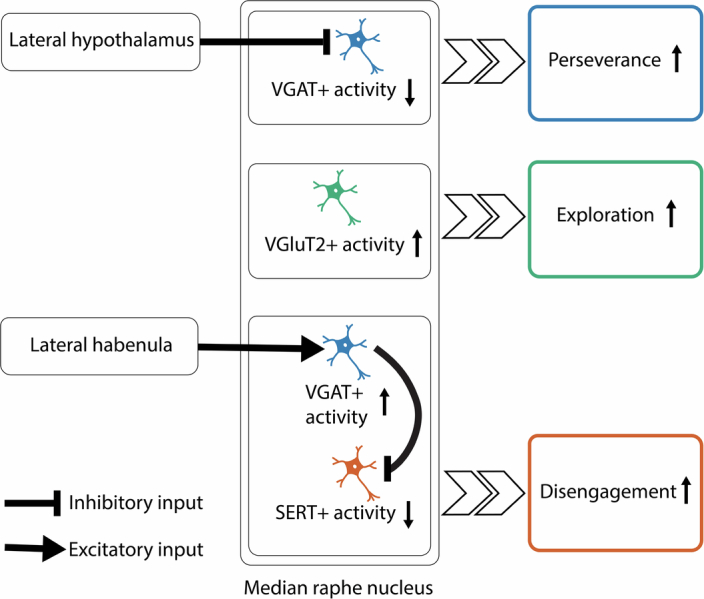
Summary of how MRN neurons affect interaction states. The schematic summarizes the findings of how MRN cell-types and their inputs from LHA and LHb regulate perseverative, exploratory and disengaged states.

Together, our data reveal unexpected brain circuits with a crucial role in regulating how mice interact with environmental resources. Three distinct MRN cell types can strongly influence decisions on whether to persevere in and exploit current or familiar options, explore alternative options or disengage from the environment (Extended Data Fig. 13). Moreover, differential engagement of MRN cell types causes switches between these behavioural strategies on a fast timescale, establishing the MRN as a central behavioural switchboard. Suppression of GABAergic neurons in MRN leads to perseverance in current goals by endowing the current, familiar option with strong positive valence. One main source of this positive valence are GABAergic neurons in LHA that inhibit MRN^VGAT^ neurons, and this inhibition is necessary for sustaining current actions or goals. Perseverance in goal-directed actions also necessitates activity of serotonergic MRN neurons that may signal emotionally positive salience. Input to the MRN from the LHb inhibits MRN^SERT^ neurons, probably via a subset of MRN^VGAT^ neurons, and conveys negative valence that decreases task engagement and suppresses sustained interactions. MRN^VGluT2^ neurons appear to operate relatively independently of these pathways, as their activation increases exploratory behaviour. Of note, suppression of MRN^VGluT2^ neurons had no effect on behaviour, indicating that activity in these neurons is not necessary for exploration. However, this pathway provides one route to actively drive exploratory choices. It remains to be determined which upstream brain areas can mediate MRN^VGluT2^ neuron activation, under which circumstances, and whether the induced exploration is goal-directed or random and value-free^40^.

Although the MRN is a strong regulator of animals’ choices, it does not act in isolation but as part of complex and distributed subcortical circuits that govern an animal’s motivations and internal states, including dopaminergic systems and the basal ganglia, the interpeduncular nucleus, DRN and others^11,13,41–45^. MRN and DRN are reciprocally connected^16,17,46,47^, and exhibit complementary serotonergic projection patterns that target distinct brain areas^46,48^. This suggests a cooperative role of MRN and DRN in balancing interaction states^13,21^. Neocortical input to MRN originates predominantly from prefrontal areas such as anterior cingulate and orbitofrontal cortex^14,15^ (Extended Data Fig. 9a,b), areas that are important for evaluation of expected costs and outcomes of different choices, and updating of value estimates while taking into account outcome uncertainty and other factors^1,2,5,8^. PFC inputs to MRN could exert cognitive control over MRN circuits, eliciting exploratory or exploitatory choices according to higher-order cost–value calculations. In particular, anterior cingulate cortex input to MRN may induce behavioural switches towards exploration through activation of MRN^VGluT2^ neurons^5,49^. How the different MRN cell types interact with each other, and the downstream circuit mechanisms of the MRN’s influence on perseverative and exploratory choices remain to be elucidated—however, all three MRN cell types have long-range projections outside the MRN^16,50^.

In summary, MRN can control either exploratory or perseverative choices, or disengagement through the differential actions of its three main cell types (Extended Data Fig. 13). Our findings establish MRN as a crucial hub for decision-making and behavioural flexibility. MRN circuits thus may also have an important role in the aetiology and possible treatment of major mental pathologies such as depressive or obsessive-compulsive disorders.

## Methods

### Animals and ethics

Mice were housed in individually ventilated cages (IVC) under controlled climate (temperature: 20–24 °C; humidity: 45–65%) in a normal light:dark cycle (12 h:12 h) with ad libitum access to laboratory food pellets and water. Wild-type C57BL/6 J (Charles River) mice, and SERT-Cre^51^ (014554, Jackson), VGAT-IRES-Cre (028862, Jackson) and VGLUT2-IRES-Cre (016963, Jackson) mice of 8–12 weeks of age from either sex were used for the experiments. We detected no influence of sex on the results, and data from male and female mice were pooled. All experimental procedures were performed in accordance with UK Home Office regulations (Animal Welfare Act of 2006), under project licence PPL PD867676F, following local ethical approval by the Sainsbury Wellcome Centre Animal Welfare Ethical Review Body. Reporting followed ARRIVE guidelines.

### Virus vector injection and optic fibre implantation

Prior to surgery, mice were subcutaneously injected with the analgesic Metacam (1 mg per kg). Mice were anaesthetized with isoflurane (5% induction, 1.2–1.8% maintenance) in oxygen (0.9 l per min flow rate). Body temperature was maintained at 36.5 °C, using a controlled heating pad. The eyes were protected from light by aluminium foil and from drying by Xailin lubricating eye ointment. Using ear bars, mice were head-fixed on a stereotactic device (Kopf, model 940) and using a scalpel blade the scalp was cut along with the midline to expose the skull. Small craniotomies were made with a dental drill (0.4 or 0.5 mm diameter) and AAV virus (60 nl) was injected into the target brain regions using a pulled glass pipette (approximately 20 µm inner diameter at the tip) and a programmable nanolitre volume injector (Nanoject III, Drummond Scientific). Fifteen minutes after the injection, the glass pipette was retracted and the incision was either sutured or glued (Vetbond), or optic fibres (for optogenetics: 200 µm diameter; for photometry: 400 µm diameter) were implanted. After cementing a custom-designed metal head plate to the skull (using light-cure Tetric EvoFlow cement (Ivoclar Vivadent), empowered by OptiBond Universal (Kerr) primer), the optic fibres were inserted 200 µm (for optogenetics) or 50 µm (for photometry) above the target brain regions and cemented to the skull. After recovery, the mice were returned to their cage. Eighteen to twenty days after the virus injection and fibre implantation surgery, mice with expression of an optogenetic opsin (ChR2 for neuronal or axonal activation, ChrimsonR for neuronal activation during photometry recording, stGtACR2^52^ for neuronal suppression, or eNpHR3.0 for axonal suppression), tdTomato (control for optogenetic stimulation) or GCaMP6f (for calcium photometry recording) were used for optogenetics/photometry experiments. After recovery, the mice were returned to their cage.

Brain regions and coordinates (from Bregma) used for virus injections: MRN (anterior–posterior (AP): −4.4 mm, medial–lateral (ML): 0.0 mm, dorsal–ventral (DV): 4.3 mm or AP: −5.5 mm, ML: 0.0 mm, DV: 4.4 mm, AP angle: 14°), LHb (AP: −1.8 mm, ML: 0.4 mm, DV: 2.7 mm) and LHA (AP: −1.6 mm, ML: 1.0 mm, DV: 5.2 mm). For optogenetic stimulation, we used AAV9-hEF1a-DIO-mCherry-hChR2 (University of Zurich; V80-9, a gift from K. Deisseroth), AAV1-CAG-hChR2-tdTomato (virus made in the host institute, using Addgene plasmid #28017 a gift from K. Svoboda), AAV9-hSyn-SIO-FusionRed-stGtACR2 (virus made in the host institute, using Addgene plasmid #105677, a gift from O. Yizhar), AAV1-Ef1a-DIO-eNpHR3.0-EYFP (Addgene #26966-AAV1, a gift from K. Deisseroth), AAV1-hSyn-eNpHR3.0-EYFP (virus made in the host institute, using Addgene plasmid #26972, a gift from K. Deisseroth), AAV1-hSyn-DIO-ChrimsonR-tdTomato (virus made in the host institute), AAV1.Syn.ChrimsonR.tdTomato (Addgene #59171-AAV1, a gift from E. Boyden) and AAV1-CAG-tdTomato (Addgene #59462-AAV1, a gift from E. Boyden), for fibre photometry recordings, AAV1-Syn-flex-GCaMP6f (virus made in the host institute, using Addgene plasmid #100833, a gift from D. Kim and GENIE Project) and for retrograde tracing experiments, retroAAV-CAG-GFP and retroAAV-CAG-tdTomato (virus made in the host institute, using Addgene plasmids #37825 and #59462, gifts from E. Boyden). Viruses for optogenetics, photometry and retrograde tracing were diluted to about 5 × 10^12^ vg ml^−1^, about 2 × 10^12^ vg ml^−1^ and about 8 × 10^12^ vg ml^−1^, respectively. For rabies virus tracing^53,54^, we first injected helper viruses AAV1-EF1a-flex-H2B-EGFP-P2A-N2cG and AAV1-EF1a-flex-EGFP-T2A-TVA (made at the host institute; diluted to about 1 × 10^13^ vg ml^−1^, 40 nl), in the target brain region (MRN), followed 10 days later by injection of rabies virus N2cG-deleted rabies-EnvA-mCherry^53^, (made at the host institute; diluted to about 1 × 10^8^ vg ml^−1^, 40 nl) at the same location. Seven days after the rabies virus injection, mice were transcardially perfused for automated serial-section two-photon imaging of the whole brain^55,56^.

### Optogenetics

Laser stimulation protocols were created and run through custom-made scripts in MATLAB or Python. For neuronal or axonal activation by ChR2 and neuronal suppression by stGtACR2 a 473 nm laser (OBIS 473 nm LX 75 mW LASER SYSTEM, Coherent), coupled to a 200 µm fibre patch cable through an achromatic fibre port (Thorlabs), was used. For axonal suppression by eNpHR3.0 and neuronal activation by ChrimsonR we used 594 nm (OBIS 594 nm LS 40 mW Laser System: Fiber Pigtail: FC, Coherent) and 647 nm (OBIS 647 nm LX 120 mW Laser System, Coherent; coupled to a 200 µm fibre patch cable through an achromatic fibre port (Thorlabs)) lasers, respectively. The laser frequency was 20 Hz (with 50% duty-cycle pulses) for depolarizing opsins (ChR2 and ChrimsonR) and 0 Hz for hyperpolarizing opsins (stGtACR2 and eNpHR3.0). The peak laser power at the tip of the fibre was ~2 mW. Using a Pulse Pal pulse generator (Open Ephys), each laser pulse for stimulating stGtACR2 was initiated and followed by a linear ramp-up and ramp-down of 500 ms, respectively. Stimulation of other opsins was done by square pulses.

### MNOI test

Mice were habituated to the experimenters and the experimental box (40 cm × 40 cm × 50 cm) every day for 3 weeks. For the last three days before the experiment, mice were also habituated to one novel object in the box to minimize stress in response to novel objects. MNOI tests were designed in a free-access manner. Mice were placed in the box for 15 min before the experiment. Then 20 novel objects (with different shapes, colours, textures and materials) were placed in random locations, and mice were allowed to interact with the objects for 2 min with optogenetic laser stimulation throughout the test duration (alternating periods of laser on 4.7 s and laser off 0.3 s). All objects were small (length between 0.5–1.0 cm) and light enough for the mice to be able to pick up and displace. Behaviour was recorded on video using one camera from the top, fixed to the ceiling of the box, and two side cameras (Raspberry Pi cameras (V2 module) with a frame rate of 25 fps).

Videos were labelled frame by frame using JAABA^57^. Analysers were blind to the experimental groups of mice. Behaviours displayed during object interaction were categorized as approaching: turning of the head towards the object accompanied by a body movement decreasing the distance between the mouse and the object; approaches ended when the mouse was at 0.5 cm distance to the object; shallow interaction: location within whisking distance of the object (closer than 0.5 cm) and facing and seemingly ‘focusing on’ the object for at least 5 frames, this can include touching the object with the snout, but not biting it; deep interaction: at least 1 of the following actions are taken: biting (taking hold of the object between its jaws and/or making nibbling motions with its head, but not walking around with the object), grabbing (holding of the object between the front paws, or standing over the object and blocking the object, effectively preventing it from moving away), and/or carrying (holding the object in its mouth and simultaneously walking around the box with it, effectively displacing the object); leaving the object: moving away from an object after shallow or deep interaction (until the nose is turned 0.5 cm away from the object). Each of these actions was attributed to one of the 20 objects. JAABA labels were imported to MATLAB for further analysis. After extracting mouse positions and movement speed, using custom-made scripts in MATLAB, times that the mouse was not performing the above actions (approaching, sniffing, deep interaction and leaving) were attributed to sitting or walking (binarizing at movement speed of 0.05 cm s^−1^). To evaluate the effect of phasic optogenetic stimulation of the MRN cell types on interaction with the novel objects, we used a 2-min MNOI test with only 5 spaced-apart novel objects. Two-second laser stimulation was applied roughly 50% of times in 1 of 3 conditions: when the mouse was not interacting with any object (at least 2 cm away from any objects or not heading towards objects), when it was within whisking distance of an object and facing the object (snout closer than 0.5 cm), and when it initiated a deep interaction with an object (biting, grabbing or carrying). The probability to switch to another object, transition probability to brief interaction (sniffing or facing towards the object within whisking distance), to deep interaction, and to no interaction, within 2 s from laser onset, was quantified as the phasic effect of laser stimulation.

### Hidden Markov model

We used a HMM^58^ to extract states that may underlie different sequences of actions. The HMM requires an estimated transition matrix, an estimated emission matrix and a sequence as input. The sequence was generated from the data of assigned mouse behaviours during the multi object interaction test. We created a non-overlapping sequence vector, with just one action happening at each time. This was achieved by creating a ranking sequence in which non-object-interacting behaviour (sitting and walking) was the lowest, then leaving, approaching, sniffing and deep interacting was the highest rank, and the highest rank was chosen as current action. We assumed that the higher the rank was, the more important information it conveyed of the mice’s state for investigatory behaviour. We set the model to three states, because we hypothesized three underlying states important for object interactions, a perseverative state in which mice persist in interacting with the current object, an exploratory state in which mice switch between multiple objects with short interactions, and a disengaged state without object interactions. As we did not have a priori information on the transitions between the states, our starting estimate for the transition matrix contained equal probabilities for all transitions, but starting with a random transition matrix did not change the results. We generated the initial estimated emission matrix by predicting the likelihood of a behaviour present in a certain state. Using a Baum-Welch algorithm^59^, we trained the model on control mice to estimate the transition and emission probabilities for the HMM (Fig. 1b). Setting the model to 4 states yielded similar results—that is, a perseverative state (mainly consisting of deep interaction), an exploratory state (mainly consisting of shallow interactions, approaching and leaving of multiple objects, with probabilities of 0.71, 0.16 and 0.11, respectively), and 2 states without object interactions. As this test was focused on object interactions, we thus chose to use the 3-state HMM. For all other groups of mice (including mice used for optogenetic and photometry experiments), the states’ probabilities at each time bin (bin size 0.5 s) were decoded from their vectors of rank-number actions based on the emission and transition matrices trained on control mice. Finally, each time bin was attributed to the state with maximum likelihood, resulting in a sequence of states. We used the built-in HMM functions hmmtrain and hmmdecode in MATLAB to acquire and evaluate the transition and emission matrices for control mice and estimate the states’ probabilities for other mice. To estimate the conditional transition probability between different states in Extended Data Fig. 1h, one time bin was used for the duration of each state, such that transition probabilities were only calculated for time points of state changes.

### Real-time place preference test

Mice with expression of an optogenetic opsin (ChR2, stGtACR2 or eNpHR3.0) or tdTomato (control) underwent a real-time place preference test in a custom-made two-chamber acrylic box (60 cm × 30 cm × 30 cm (l × w × h)). After 10 min of habituation to the box, one of the two chambers was paired with laser stimulation (triggered by entering the chamber). The laser-coupled chamber was randomly assigned. The total test duration was 10 min. Mouse behaviour was analysed using Bonsai software (https://bonsai-rx.org/). Using a custom-made MATLAB script, the preference for the opto-linked chamber was calculated as follows: 100% × (duration of time spent in the opto-linked chamber − duration of time spent in the non-stimulation chamber)/total time).

### Self-stimulation test

Mice with expression of an optogenetic opsin (ChR2, stGtACR2 or eNpHR3.0) or tdTomato (control) were habituated for 1 h to a custom-made experimental box (40 cm × 40 cm × 50 cm; *l* × *w* × *h*) with a two-port nose-poke system one day before the test. During the habituation period, drops of 10% sucrose water were delivered through both ports to habituate the mice to the nose-poke ports. No sucrose water was delivered during the test. Instead, an infrared sensor in only one nose-poke port triggered optogenetic stimulation when the mouse entered the port with its nose, and stimulation continued throughout the time period the mouse’s nose remained in the nose-poke port. An infrared sensor in the other nose-poke port did not trigger any stimulation. The test lasted 1 h. The number of returns to each port was detected by the sensors (connected to an Arduino UNO microcontroller board) and preference for returning to the opto-linked nose-poke port was calculated as follows: 100% × (number entries into the opto-linked nose-poke port − number of entries into the non-stimulation nose-poke port)/(number of entries into the opto-linked nose-poke port + number of entries into the non-stimulation nose-poke port). The length of time mice spent with their snout in a port was not taken into account for this analysis.

### T-maze tests

To measure the effects of optogenetic stimulation on exploratory versus perseverative choices based on acquired choice-outcome knowledge, we trained food-restricted mice on a T-maze task^60,61^, in which a food reward (10 mg of their preferred food, yoghurt drop or their regular food (Teklad Global 2016; 16% protein, 4% fat)) was provided consistently only in one specific arm and not the other. Training started after 2 days of 15-min habituation to the T-maze, to the experimenter and to a soft towel by which they were placed in and picked up from the T-maze. Each training trial started with placement of the mouse at the start of the central arm and ended with the mouse turning into either of the choice arms (left or right). At the end of each trial the entrance of the choice arms was blocked to prevent the mouse from returning to the other arms. After reaching the end of the non-rewarded arm or after eating the food reward at the end of the rewarded arm, mice were gently picked up in the soft towel and placed back at the start of the central arm. After mice achieved more than 90% correct trials in two sequential 50-trial sessions (well-trained; mean ± s.d.: 7.42 ± 2.66 sessions), they entered one of the two tests. For behaviour sessions with optogenetic manipulations (55 trials), optogenetic stimulation was delivered in the central arm of the T-maze until after mice chose one of the arms. In these sessions, food reward was either provided in the same arm as during training, in which case the percentage of trials in which they entered the non-rewarded arm indicated their tendency to explore, or the reward location in the T-maze was reversed—that is, the previously rewarded arm was not rewarded anymore. In this second scenario, normally mice start exploring the other, previously non-rewarded arm and eventually learn to expect reward only at this location. The percentage of trials in which they entered the previously rewarded arm (that is, the currently non-rewarded arm) indicated their tendency to persevere in the previously rewarded choice.

### Nose poke–reward association task

To measure the effect of optogenetic stimulation on task engagement, we used a previously described nose poke–reward association task^62^. After three days of water restriction, mice with expression of an optogenetic opsin (ChR2, stGtACR2 or eNpHR3.0) or tdTomato (control) were trained to enter their nose into a nose-poke port on one wall of a custom-made operant chamber (25 cm × 20 cm × 30 cm (*l* × *w* × *h*); in a sound-attenuating box) in order to receive a water reward from a lick spout on the wall opposite to the nose-poke port. At the start of each trial, the nose-poke port was illuminated. Upon completion of a successful nose-poke, the light in the nose-poke port was turned off, and a white noise sound was turned on to indicate the availability of reward. A water reward was delivered to the lick spout, via a solenoid valve, upon licking. A nose-poke entry followed by a lick was considered a single completed trial. Mice were free to run back and forth between the nose-poke port and the reward spout and complete trials at their own pace. Training continued daily until mice were able to complete more than 85 trials per session in two sequential daily 30-min sessions (mean ± s.d.: 10.40 ± 4.35 sessions). Following training, mice performed the same task during which they received 3 epochs of 2-min optogenetic stimulation with random off-stimulation time intervals (not less than 2 min) between 2 min and 20 min from the session’s onset. The number of completed trials indicated the engagement level. Data acquisition and stimulation was performed using a Pycontrol state machine (https://pycontrol.readthedocs.io/en/latest/).

### Nose poke–reward association task with distractor nose-poke ports

To measure the effect of optogenetic stimulation on evoking exploration, we modified the nose poke–reward association task, by adding two nose-poke ports without function at the side walls. Following training in the standard nose poke–reward association task, mice with expression of ChR2 in VGluT2^+^ or VGAT^+^ neurons in MRN, or tdTomato (control) mice were tested in the presence of the two additional nose-poke ports, used as distractors to motivate exploration. Only an entry into the previously learned nose-poke port followed by a lick from the lick spout resulted in water-reward delivery. At the beginning of the test, mice were given a 5-min period to explore all the nose-poke ports and understand that the additional nose-poke ports are not associated with reward. During the following 20 min, mice received optogenetic stimulation (for a random time period between 1 and 2 s) in 25–35% of trials upon entering into a 0.5 cm radius around the reward-associated nose-poke port. From laser onset until completion of the trial (receiving a water reward), the number of times mice interacted with the distractor nose-poke ports or the lick spout (without initiating the trial) was counted as number of interactions with distractors in this trial. Data acquisition and stimulation was performed using a Pycontrol state machine and data analysis was performed by a custom-made MATLAB script.

### Three-armed bandit task

To measure the effect of optogenetic manipulation of MRN^VGluT2^ and MRN^VGAT^ neurons more specifically on the trade-off between exploration and exploitation, we used a 3-armed bandit task with 3 water lick ports, P1–3, on 3 walls of a custom-made pentagon-shaped operant chamber. Reward probability at P3 was constant at 50%, and reward probabilities at P1 and P2 fluctuated in blocks (25–35 trials), between 10% and 90% (for VGluT2 neuron activation) or between 25% and 75% (for VGAT neuron suppression). Each trial was self-initiated by licking any of the three lick ports, followed by a water reward or no-reward upon licking, and a 2-s inter-trial-interval.

Mice were trained daily until they had learned to switch between the high-probability ports—that is, chose the changing high-probability port in more than 85% of trials for 90%/10% reward probabilities, or in more than 70% of trials for 75%/25% reward probabilities in each block (within the first 20 min, when mice were more engaged) during two consecutive 30-min sessions. Following training, mice performed several sessions of the same task with and without laser stimulation. During the laser stimulation sessions, in 2-3 blocks (within the first 20 min), mice received one epoch of optogenetic stimulation. For VGAT suppression, laser stimulation started after the first 3 consecutive trials of the mouse choosing the new high-probability port after the start of the block. The laser was on until the end of the current block. For VGluT2 activation, laser stimulation started after the 5 consecutive trials of the mouse choosing the new high-probability port after the start of the block, and lasted for 2 s. Behaviour in the interspersed sessions without optogenetic manipulations was used as control data. Data acquisition and stimulation was performed using a Pycontrol state machine and data analysis was performed by a custom-made MATLAB script.

### TMT aversion test

Mice were habituated to the experimenter for three days, 30 min a day, and to the experimental box (40 cm × 40 cm × 50 cm (*l* × *w* × *h*)) for 30 min the day prior to the experiment. For the test, mice were habituated in the box for 15 min, after which a small object covered with 3 µl trimethylthiazoline (TMT, BioSRQ: purity >90.0%), a constituent of fox urine, was placed inside the box for 2 min. Optogenetic stimulation was applied for the 2-min duration, with repeated pulse trains of 4.7 s laser on and 0.3 s laser off (to avoid overstimulation of neurons and tissue damage by heat accumulation). Mouse behaviour was recorded (on video (Raspberry Pi camera (V2 module); with 25 fps) and videos were labelled frame by frame using JAABA^1^. Analysers were blind to the experimental groups of mice. Behaviours displayed during the test were categorized as: approaching (approaching towards the object, from start of body movement until reaching proximity of 0.5 cm), interacting (sniffing, grabbing, carrying or biting the object) and retreating (moving away, upon reaching the object, in the opposite direction to the approach). JAABA labels were imported to MATLAB for further analysis. In addition, mouse position and movement speed were extracted using custom-made MATLAB scripts. A retreat upon reaching the TMT object was counted as an escape if the average speed of the retreat exceeded 0.5 cm s^−1^ within the first 1 s. Varying the speed threshold (0.3, 0.4, 0.5 or 0.6 cm s^−1^) did not influence the results. Escape probability was calculated as the number of approaches leading to escape divided by the total number of approaches.

### Visually evoked fear response test

Mice with expression of ChR2 in VGluT2^+^ MRN neurons underwent a visually evoked fear response test to see whether activation of these neurons changes their fear response to an innately threatening visual stimulus, an overhead expanding—that is, looming—black disc^63^. Experiments assessing escape behaviour in response to looming stimuli were performed in a custom-made transparent acrylic arena (80 cm × 26 cm × 40 cm (*l* × *w* × *h*)) with a red-tinted acrylic shelter (14 cm × 14 cm × 14 cm (*l* × *w* × *h*)) placed on one end (safe zone) and overhead looming stimuli presented on the other end (threat zone)^64,65^. The arena was placed in a large light-proofed and sound-attenuating box with a near-IR GigE camera (acA1300-60 gmNIR, Basler; with a frame rate of 50 fps) fixed on the ceiling to video record the behaviour. To display looming stimuli, a projector (HF85JA, LG) was mounted in the box and back-projected via a mirror onto a suspended horizontal screen (60 cm above arena floor, 100 cm × 80 cm; ‘100 micron drafting film’, Elmstock). The screen was kept at a constant background luminance level of 9 cd m^−2^. The arena was illuminated by four infrared LEDs to ease tracking of the mice. Video recording and optogenetics laser stimulations were triggered through Bonsai (https://bonsai-rx.org/). Mice were placed in the arena 20 min before the test to habituate to the new environment. Stimulation was triggered manually by a keyboard when the mouse reached the threat zone. The stimulus was only triggered when the mouse was facing and walking toward the threat zone, with an inter-stimulus interval of at least 2 min. Each visual stimulation consisted of three consecutive looming stimuli (expanding black spot at a linear rate of 55° s^−1^) in a 3-s period. Visual stimuli of 10%, 50% and 90% contrasts were presented in a randomized order. Laser and non-laser trials of the same stimulus contrast were always presented as paired trials, in a randomized but consecutive order (10 repetitions × 3 contrasts × 2 laser conditions). Optogenetic stimulation in laser trials started 0.5 s before the visual stimulus. Position and running speed of the centre of the mouse were processed using a custom-made MATLAB script. A successful escape was defined as a return to the shelter with an average running speed higher than 0.4 cm s^−1^ (varying this threshold to 0.3 or 0.5 cm s^−1^ did not change the significance of the results) in the 2 s after the stimulus onset and reaching the shelter within 5 s of stimulus onset.

### Sucrose preference test

The sucrose preference test is frequently used to measure anhedonia in mice based on a two-bottle choice paradigm^66,67^. A decreased responsiveness to rewarding sucrose compared to a control group indicates anhedonia. On the first day of habituation, mice with expression of ChR2 in LHb input to MRN and control mice were provided with 2 identical bottles of water and on the second day with 2 identical bottles of 1% sucrose water for 24 h, respectively. During the next 4 days, mice were provided with 2 bottles of water, while they were receiving repeated optogenetic stimulation for 24 h (60 s laser on, every 240 s). The next day, after 12 h of water deprivation, mice were tested with 1 bottle of water and 1 bottle of 1% sucrose water (for 12 h), without optogenetic stimulation. Sucrose preference was calculated by the percentage of sucrose water consumed relative to the total liquid consumption.

### Arousal level measures and analysis

For these experiments, mice were habituated for three days (30 min a day) to be head-fixed on a styrofoam running wheel (16 cm diameter, 12 cm width) in a sound-attenuating box with dim ambient light. Mice with expression of an optogenetic opsin (ChR2, stGtACR2 or eNpHR3.0) or tdTomato (controls) were used for measuring changes in physiological arousal level caused by optogenetic stimulation. Mice were head-fixed and an infrared LED light was directed to the face to illuminate the pupil (mounted 50 cm away). Their running speed was recorded using a rotary encoder (Kubler Encoder 1000 ppr) coupled to the wheel axle. Mice received optogenetic stimulation for 3-s periods (30 trials with 12-s intervals). The effect of optogenetic stimulation on the arousal level was monitored by recording videos of the pupil and whiskers (using two cameras (22BUC03, ImagingSource) with frame rates of 30 fps), as well as the running speed. Whisker activity during each frame was calculated as the absolute difference between the colour intensity of each pixel of this frame and the previous frame, averaged over all pixels. Baseline *z*-scored pupil size, whisker activity and running speed were determined using custom-made scripts in MATLAB.

### Calcium photometry recordings and data analysis

Mice were habituated to the experimenters for three days (30 min a day) and to the experimental box (40 cm × 40 cm × 40 cm) for three more days (15 min a day). Three weeks after the GCaMP6 viral injection and the optic fibre implantation, calcium activity of MRN neurons, LHb axons or LHA^VGAT^ axons in MRN was recorded in freely moving mice. Recording from MRN^SERT^ neurons was performed in three different conditions, in the presence of multiple novel objects, of food pellets, or of TMT objects. Recordings from MRN^VGAT^ neurons, MRN^VGluT2^ neurons, LHb axons in MRN and LHA^VGAT^ axons in MRN were done in the presence of multiple novel objects. Movies of mouse behaviour and fluorescence were recorded simultaneously using Raspberry Pi cameras (V2 module, frame rate: 25 fps) and a fibre photometry system (optics from Doric Lenses; acquisition board and recording software from PyPhotometry (https://pyphotometry.readthedocs.io/en/latest); sampling rate: 130 Hz), respectively. Excitation lights at the tip of the optic fibre were adjusted to around 30 µW. The data for fibre photometry were analysed using a custom-made MATLAB program. A linear drift correction was applied to raw signals of calcium-dependent (GCaMP excited at 473 nm) and isosbestic fluorescence (GCaMP excited with 405 nm light) to correct for slow changes such as photobleaching. To correct for non-calcium-dependent signals and artifacts, the isosbestic fluorescence trace was linearly fitted to the calcium-dependent GCaMP fluorescent signal and subtracted, providing a measure of relative transient changes in fluorescence (d*F*/*F*). Mean baseline was taken as the average d*F*/*F* signal of the entire recording session. Subsequently, *z*-scored d*F*/*F* was calculated by subtracting the mean baseline and dividing by the standard deviation of the baseline distribution. Behaviours were analysed using JAABA and MATLAB, similar to experiments with optogenetic stimulation. Data points in Figs. 1g and 2e are *z*-scored d*F*/*F* values averaged over 3 s after the onset of behaviours stated in the figure legends. Data points in bar graphs in Figs. 1h and 2f are *z*-scored d*F*/*F* values averaged over the time course of the associated interaction states.

### Electrophysiological recordings and data analysis

To compare the effect of optogenetic activation of LHb and LHA^VGAT^ inputs to MRN on MRN and VTA neurons, we performed acute electrophysiological single-unit recordings from head-fixed mice while activating the MRN inputs. To this end, we expressed ChR2 in LHb (in 3 C57BL/6 mice) or LHA^VGAT^ neurons (in 3 VGAT-Cre mice), cemented a metal head plate and implanted a fibre above the MRN (as described above). Around three weeks after surgery, mice were habituated to the head-fixation and electrophysiology setup for 3 days, before one acute recording session. A high-density electrophysiology probe (Neuropixels 1.0 prototype 3 A) was inserted to record from MRN (AP: 4.4 mm, ML: 0.1 mm) and posterior VTA (AP: 3.6 mm, ML: 0.5 mm) (through craniotomies above them and based on the distance from the surface of the brain; MRN depth: 3.8–4.8 mm and VTA depth: 3.9–4.6 mm). Prior to insertion, the probe was coated with DiI (1 mM in isopropanol, Invitrogen) for post hoc histological alignment. The probe was inserted using a micromanipulator (Sensapex). Using a Pulse Pal pulse generator (Open Ephys) a train of 100–200 laser pulses of 10 ms for activation of LHb input to MRN or 100 ms for activation of LHA^VGAT^ input to MRN with random inter-stimulus-intervals was used, while recording from the MRN or VTA. Data were acquired using spikeGLX (https://github.com/billkarsh/SpikeGLX, Janelia Research Campus), high-pass filtered (300 Hz) and sampled at 30 kHz.

Spikes were sorted with Kilosort2 (https://github.com/cortex-lab/Kilosort) and Phy^68^. Each unit was attributed to the channel with the highest waveform amplitude. A single unit was considered to have a robust direct input from LHb if more than 90% of laser pulses resulted in an increase in its firing rate within 10 ms from the laser onset (compared to its firing rate in a 100 ms time window before the laser onset). A single unit was considered to have a robust direct inhibitory input from LHA, if more than 90% of laser pulses resulted in a decrease in its firing rate within 100 ms from the laser onset (compared to its firing rate in a 100 ms time window before the laser onset).

### Multi-colour single-molecule mRNA in situ hybridization

In situ hybridization was performed using RNAscope technology. To quantify the overlap between MRN cell types (for Extended Data Fig. 1a,b), we used C57BL/6 mice. To evaluate specificity of Cre expression in MRN of SERT-Cre mice (for Extended Data Fig. 1c–e) we expressed eGFP in Cre-expressing MRN cells through injection of AAV-syn-flox-eGFP into the MRN two weeks before brain extraction). After induction of deep anaesthesia by isoflurane (5%), brains were extracted and immediately fresh-frozen in optimal cutting temperature (OCT). Using a cryostat, brains were sliced into 15-µm sections, mounted on glass slides, and stored at −80 °C. Multi-fluorescence mRNA in situ hybridization was performed using ACDBio RNAscope multiplex fluorescence V2 assay (https://acdbio.com/rnascope-multiplex-fluorescent-v2-assay). The RNAscope protocol was carried out as indicated in the user manual of the ACDBio RNAscope. Brain sections of the MRN were post-fixed with 4% chilled paraformaldehyde (PFA) in PBS for 60 min and then dehydrated through 4 dehydration steps in 50%, 70%, 100% and 100% ethanol, respectively, at room temperature. After air drying for 10 min at room temperature, Protease IV was applied to the slices for 15 min at room temperature. Then, they were washed out three times by rinsing in phosphate-buffered saline (PBS). VGluT2-C1 (1170921-C1), SERT-C2 (315851-C2) and VGAT-C3 (319191-C3) (for Extended Data Fig. 1a,b) or SERT-C2 (315851-C2) and eGFP-C3 (538851-C3) (for Extended Data Fig. 1c–e) were pipetted onto each slice. Probe hybridization took place in an oven set to 40 °C for 2 h, and then, slices were rinsed in 1× wash buffer. After amplification and fluorophore labelling steps, slices were mounted with DAPI (4′,6-diamidino-2-phenylindole) vector shield. Immediately after mounting, the stained slices were imaged by confocal SP8 microscope (Leica) using a 20× objective. For quantification of numbers of labelled and co-labelled cells we used ImageJ (Fiji).

### Histology and microscopy

For determining inputs and outputs of specific brain areas, and for histological confirmation of injection sites and fibre locations, at the end of experiments mice were euthanized by an overdose of pentobarbital (intraperitoneal injection, 80 mg kg^−1^) and transcardially perfused (0.01 M PBS, followed by 4% PFA in PBS). After extraction, brains were post-fixed by 4% PFA solution overnight and consequently embedded in 5% agarose (A9539, Sigma). Imaging was performed using a custom-built automated serial-section two-photon microscope^55,56^. Coronal slices were cut at a thickness of 40 μm, and images were acquired from 2–8 optical planes (every 5–20 μm) with approximately 2.3 μm per pixel resolution. Scanning and image acquisition were controlled by ScanImage^69^ v5.5 (Vidrio Technologies) using a custom software wrapper for defining the imaging parameters (https://zenodo.org/record/3631609). Cell detection and counting was performed using Cellfinder (https://github.com/brainglobe/cellfinder). Post hoc histological alignment of the DiI-coated electrophysiology probes was performed by registering 3D images of brains to a reference brain atlas (https://mouse.brain-map.org/static/atlas) using Brainreg (https://brainglobe.info/documentation/brainreg/).

### Overall experimental design and analysis

No statistical methods were used to predetermine sample sizes, but our sample sizes were determined based on previous studies^70,71^. The order of mice in different experimental groups was randomly assigned. Experimenters were not blind to the experimental conditions, but the collected data were encoded blindly and analysers were blind to experimental condition. All data were analysed using JAABA, MATLAB, Python and Bonsai.

### Statistical analysis

Data are represented as median ± bootstrap standard error, unless stated otherwise. All statistical analyses were performed using InVivoTools MATLAB toolbox^72–74^ (https://github.com/heimel/InVivoTools) or custom-made MATLAB scripts. First, normality of the data distribution was tested, using a Shapiro–Wilk normality test. To assess group statistical significance, if data were normally distributed we used parametric tests (*t*-test and paired *t*-test for non-paired and paired comparisons, respectively), and otherwise non-parametric tests (Mann–Whitney *U*-test and Wilcoxon signed-rank test for non-paired and paired comparisons, respectively), followed by a Bonferroni *P* value correction for multi group comparisons. For multi group comparisons with subgroups within groups we used nested one-way ANOVA. To evaluate optogenetic effect over multiple trials in the 3-armed bandit task we used 2-way repeated measures ANOVA. To statistically compare distributions of discrete data across different groups, chi-square test was used. Individual data points are shown in the figures. Statistics used in the main figures are listed in Extended Data Table 1 and statistics in Extended Data figures are listed in the figure legends.

### Reporting summary

Further information on research design is available in the Nature Portfolio Reporting Summary linked to this article.

## Online content

Any methods, additional references, Nature Portfolio reporting summaries, source data, extended data, supplementary information, acknowledgements, peer review information; details of author contributions and competing interests; and statements of data and code availability are available at 10.1038/s41586-025-08672-1.

## Supplementary information

Reporting Summary

Supplementary Video 1 **A control mouse in the MNOI test**. Top right square indicates the behavioural state extracted by the HMM; orange: disengaged state; blue: perseverative state; green: exploratory state

Supplementary Video 2 **Suppressing VGAT**^**+**^ **MRN neurons during the MNOI test**. Top right square indicates the behavioural state; orange: disengaged state; blue: perseverative state; green: exploratory state

Supplementary Video 3 **Activating** **VGluT2**^**+**^ **MRN neurons during the MNOI test**. Top right square indicates the behavioural state; orange: disengaged state; blue: perseverative state; green: exploratory state

Supplementary Video 4 **A control mouse in the TMT aversion test**

Supplementary Video 5 **Suppressing VGAT**^**+**^ **MRN neurons during the TMT aversion test**

Supplementary Video 6 **Suppressing SERT**^**+**^ **MRN neurons during the nose-poke reward association task**

Supplementary Video 7 **Activating LHb input to MRN during the nose-poke reward association task**.

Supplementary Video 8 **Activating GABAergic LHA input to MRN during the TMT aversion test**

Supplementary Video 9 **Activating GABAergic LHA input to MRN during the MNOI test**. Top right square indicates the behavioural state; orange: disengaged state; blue: perseverative state; green: exploratory state

Supplementary Video 10 **Suppressing GABAergic LHA input to MRN in a MNOI test**. Top right square indicates the behavioural state; orange: disengaged state; blue: perseverative state; green: exploratory state

## Source data

Source Data Fig. 1

Source Data Fig. 2

Source Data Fig. 3

Source Data Fig. 4

Source Data Fig. 5

Source Data Fig. 6

## Figures and Tables

**Extended Data Table 1:**
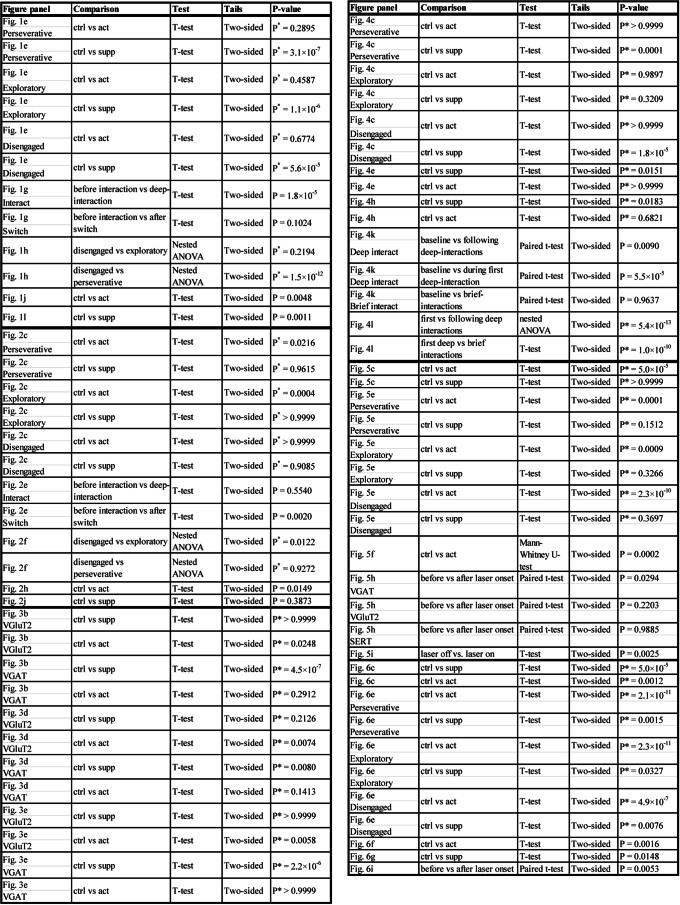
Table of statistics of the main figures

*: Bonferroni corrected p-value.

## Data Availability

The data that support the findings of this study are available from the corresponding authors upon reasonable request. Source data are provided with this paper, as well as at https://github.com/mehranahmadlou/Interaction-States.

## Code availability

Data analysis codes of this study are available at https://github.com/mehranahmadlou/Interaction-States.
